# Probiotic effect of *B. subtilis* PS-216 in broiler chickens: modulation of weight, feed conversion, short chain fatty acids, microbiota and meat quality

**DOI:** 10.1186/s42523-025-00489-7

**Published:** 2025-11-19

**Authors:** Katarina Šimunović, Vida Rezar, Polonca Štefanič, Luka Lipoglavšek, Lijana Fenedl, Uroš Krapež, Tatjana Pirman, Gorazd Avguštin, Janez Salobir, Olga Zorman Rojs, Ines Mandić Mulec

**Affiliations:** 1 Department of Microbiology, Biotechnical Faculty, University of Ljubljana, Večna pot 111, Ljubljana, Slovenia; 2 Department of Animal Science, Biotechnical Faculty, University of Ljubljana, Groblje 3, Domžale, Slovenia; 3 Institute for Poultry, Birds, Small Mammals and Reptiles, Veterinary Faculty, University of Ljubljana, Gerbičeva 60, Ljubljana, Slovenia

**Keywords:** *Bacillus subtilis* PS-216, Broiler chicken probiotic, Microbiota modulation, Feed conversion, Meat quality

## Abstract

*Bacillus*-based probiotics are recognized as safe and effective alternatives to antibiotic growth promoters in poultry production. This study evaluated the effects of *Bacillus subtilis* PS-216 on broiler performance, gut health, and meat quality. Broilers received PS-216 spores continuously via drinking water (2 × 10^9^ spores/L; SW) or feed at low (2 × 10^6^ spores/kg; SF1) or high (2 × 10^9^ spores/kg; SF2) concentrations. Parameters were assessed at days 23 and 44, including growth performance, lymphatic organ weights, cecal microbiota composition, short-chain fatty acid (SCFA) profiles, and meat quality. All treatment groups demonstrated significant performance improvements compared to controls: body weight increased by 7.4–8.1% and feed conversion ratio improved by 3–11%. Thymus weight increased by approx. 22% across all groups, indicating immunomodulatory effects. Microbiota analysis revealed beneficial shifts including increased butyrate-producing taxa (*Selenomonadales*, *Clostridium* sensu stricto) and reduced stress-associated *Anaeroplasma*. The SF2 group showed enhanced cecal butyrate and acetate concentrations at day 23, with sustained butyrate elevation at day 44. Additionally, meat quality improvements were observed, particularly reduced electrical conductivity and improved breast meat color distribution in the SF2 group. Feed supplementation at 2 × 10^9^ spores/kg (SF2) provided the most comprehensive benefits across all measured parameters. These findings demonstrate that *B. subtilis* PS-216 enhances broiler performance through beneficial microbiota modulation and immune function improvement, supporting its potential as a sustainable alternative to antibiotic growth promoters in commercial poultry production.

**Supplementary Information:**

The online version contains supplementary material available at 10.1186/s42523-025-00489-7.

## Introduction

The increasing prevalence of antimicrobial resistance (AMR) poses a critical global health threat, resulting in infections that are difficult or impossible to treat in both animals and humans. The prophylactic use of antibiotics as growth promoters in livestock production has been identified as a major contributor to the development and spread of AMR [1, 2]. This growing recognition of AMR risks has prompted widespread regulatory restrictions on the prophylactic use of antibiotics in livestock globally. The European Union led these efforts with a comprehensive ban implemented in 2006 under Regulation 1831/2003/EC on additives for use in animal nutrition and further in 2019 with Regulation 2019/4 on medicated feed and Regulation 2019/6 on veterinary medicinal products. The United States subsequently adopted parallel measures in 2017, guided by the FDA’s Guidance for Industry (GFI) #213, with numerous other countries implementing similar restrictions.

Historically, antibiotic supplementation in livestock, including poultry, has provided substantial benefits including improved growth rates and weight gain, enhanced feed conversion efficiency, and reduced incidence of infectious diseases [3, 4]However, the regulatory restrictions on antibiotic use as feed additives, combined with increasing consumer demand for food safety, sustainability, and security, have intensified the search for alternative strategies to maintain animal health and performance. Probiotics represent a promising and sustainable pre-harvest intervention strategy for performance enhancement in modern poultry production [5, 6].

Common probiotics used in the poultry industry include yeasts such as *Saccharomyces* spp., and *Candida* spp., and bacteria such as *Lactobacillus* spp., *Bifidobacterium* spp., *Lactococcus* spp., *Streptococcus* spp., and *Bacillus* spp [4]. Among these, *Bacillus*-based probiotics demonstrate distinct advantages due to their ability to form highly resilient spores. These spore-forming characteristics confer strong resistance to harsh conditions encountered during feed processing, including high temperatures and pressure during pelleting procedures. Additionally, *Bacillus*-based probiotics offer extended shelf life, potentially up to five years, and maintain high viability throughout transit through the gastrointestinal tract, ensuring effective colonization and sustained probiotic activity. Furthermore, these spore-forming probiotics exhibit immunomodulatory properties and contribute to the environmental sustainability of poultry production systems [4, 7–9].

*Bacillus*-based probiotics have demonstrated efficacy in enhancing poultry performance through increased body weight (BW) and improved feed conversion ratio (FCR). These benefits are mediated through improved gut health, characterized by enhanced villus elongation, increased crypt depth, and reduced intestinal lesions in challenged animals. Through modulation of intestinal microbiota composition, probiotics facilitate beneficial short-chain fatty acid (SCFA) production, particularly butyrate, while simultaneously reducing pathogenic microorganisms, thereby improving both animal health and food quality and safety [7, 8, 10]. Commercial formulations containing *B. subtilis*, *B. amyloliquefaciens*, *B. licheniformis*, and *B. cereus* var. *toyoi* are currently utilized in the poultry industry as single-strain, multi-strain, or multispecies probiotic preparations. However, their effectiveness demonstrates considerable variability depending on specific species and strain characteristics [8].

The strain *B. subtilis* PS-216 was isolated from sandy soil along the banks of the river Sava in Slovenia [11]. This strain has demonstrated potent inhibitory activity against *Campylobacter jejuni*, a major foodborne pathogen prevalent in poultry production environments [12, 13], and exhibits the ability to effectively eliminate preformed *C. jejuni* biofilms under in vitro conditions [14]. In a previous in vivo challenge study, continuous administration of *B. subtilis* PS-216 in drinking water at a concentration of 2 × 10^9^ CFU/L significantly reduced *C. jejuni* colonization in the ceca of broiler chickens [13]. While the antimicrobial effects of *B. subtilis* PS-216 against poultry associated foodborne pathogens, including *C. jejuni* and *Salmonella enterica*, have been extensively documented through in vitro studies [12–18], its broader physiological impact on the chicken host remains largely unexplored beyond preliminary observations of growth promotion during early rearing stages.

The objective of the present study was to comprehensively characterize the probiotic effects of *B. subtilis* PS-216 in broiler chickens. Specifically, we investigated its impact on growth performance, gut health parameters, immune function, cecal microbiota composition, short-chain fatty acid profiles, and meat quality characteristics, providing a comprehensive evaluation of its potential benefits for sustainable poultry production systems.

## Materials and methods

### *B. subtilis* PS-216 spore preparation

*B. subtilis* PS-216 spores were prepared as described previously [13] with some modifications. Briefly, the spore production medium (16 g/L Nutrient broth (Biolife, Milano, Italy), 2 g/L KCl (Sigma Aldrich, Darmstadt, Germany), 1 mM MgSO_4_ × 7H_2_O (Sigma Aldrich, Darmstadt, Germany), 1 mM Ca(NO_3_)_2_ × 4H_2_O (Sigma Aldrich, Darmstadt, Germany), 1 µM FeSO_4_ × 7H_2_O (Sigma Aldrich, Darmstadt, Germany), 10 µM MnCl_2_ (Sigma Aldrich, Darmstadt, Germany), 2.8 mM glucose (Sigma Aldrich, Darmstadt, Germany), 2.8 mM D-ribose (Sigma Aldrich, Darmstadt, Germany) was inoculated with 1% of PS-216 overnight culture (LB broth; Lennox, Condalab, Madrid, Spain), 37 °C, 16 h with shaking at 200 rpm) and further incubated for 4 days (37 °C, 200 rpm). After incubation the culture was treated at 80 °C for 30 min, centrifuged (10,000 *g*, 10 min) and washed with saline solution three times. The pellet containing only spores was frozen at -80 °C for at least 24 h and freeze-dried for 3 days. The dry powder was mixed with feed to obtain the appropriate number of spores prior to use.

The number of spores was monitored in all feed and water samples as well as in the cecum content. Samples were treated at 80 °C for 30 min. Serial dilutions of treated samples were spread onto *Bacillus* Chromo Select Agar (Sigma Aldrich, Darmstadt, Germany) and the spore count per ml of water, g of feed, and cecum content was determined in the samples.

### Animal ethics

The animal experiment was approved by the Animal Ethics Committee of the Administration for Food Safety, Veterinary Sector and Plant Protection of the Republic of Slovenia (U34401-6/2023/9).

### Broilers, housing, and dietary treatments

One-day-old male Ross 308 Aviagen^®^ broilers (*n* = 168) were randomly assigned to 12 deep litter pens, with four dietary treatments (3 pens per treatment type). In the experimental room with 12 floor pens, 3 replicates per group and 14 birds per replicate pen were used. Each pen (0.95 m × 1.26 m = 1.20 m^2^) with sawdust as a litter was equipped with a plastic feeder and an automatic watering system with five nipple drinkers except three pens where the nipple systems were blocked and water was provided through bell drinkers. Experimental feed in a mashed form and fresh water were provided ad libitum throughout the trial. Experimental groups were divided by treatment regime into the (i) Control groups, no addition of spores, (ii) Spores added to drinking water (in bell drinkers) at a concentration of ~ 2 × 10^9^ spores/L water (SW, comparable to Šimunović et al.^1 [3]^), (iii) Spores added to feed in a low concentration of ~ 2 × 10^6^ spores/kg of feed (SF1), and (iv) Spores added to feed in a concentration of ~ 2 × 10^9^ spores/kg of feed (SF2).

Broilers were reared under a standard lighting program of 23 L:1D during the first week, and 18 L:6D from 8 d to the end of the experiment (day 44). Upon arrival, birds were individually marked. Body weight (BW) was recorded weekly throughout the experiment at slaughter to determine the chicken BW per unit and average BW per experimental group. Average daily feed intake (ADFI), body weight gain (BWG), and feed conversion ratio (FCR) were recorded to determine broiler growth performance. The experimental diets were formulated and fed in two phases: during the first 15 days, chickens were fed a starter diet; from day 16 to the end of the experiment (day 44), a finisher diet. The experimental diets were formulated according to the nutritional specifications and recommendations for Ross 308 broilers [19]. Components and calculated energy and nutrient contents of the starter and finisher feed formulations are listed in Table S1. Samples of finisher diets were collected to determine proximate and mineral content (Table S2 and Table S3).

### Sample collection

**Fig. 1:**
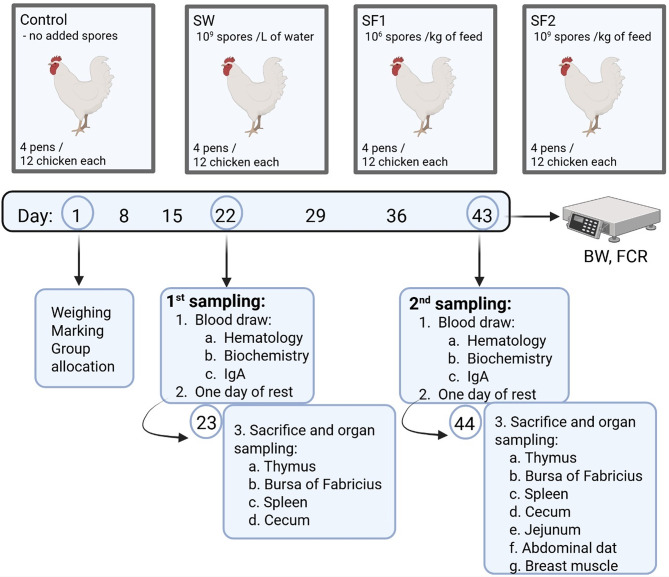
Experimental setup and sampling design. Figure has been generated using BioRender (app.biorender.com)

For sampling procedures four broilers were randomly selected from each pen (12 broilers per treatment type) and marked. After blood collection, selected broilers were returned into pens for 24 h rest. The next day broilers were euthanized by cervical dislocation and samples of the thymus, bursa of Fabricius, spleen, jejunum and cecum were collected. Samples were collected at two sampling points. The first sampling was performed at day 22 (day 23) and the second sampling at day 43 (day 44). Additionally, at the second sampling (day 44) abdominal fat and breast muscle samples were collected (Fig. 1).

Blood was collected from the brachial wing vein using a 25-gauge, 1-inch-long needle. Samples were drawn into EDTA-containing tubes (for hematology) and serum separator tubes (for routine biochemistry and IgA ELISA). Samples for routine hematology and biochemistry were analyzed within 1 h after sampling. The day after blood collection, broilers were weighed and euthanized by cervical dislocation. Immediately after euthanasia the thymus, bursa of Fabricius, spleen and ceca were removed and weighed. Each pair of ceca was placed into sterile plastic tubes, promptly refrigerated (for a maximum of 30 min), and then frozen at -80 °C until further analysis. For morphometric analysis, approximately 5 cm long segments of jejunum were sampled anterior to the Meckel’s diverticulum. The collected segments were first flushed with 0.9% saline to remove intestinal contents and then fixed in 10% buffered formalin solution.

### Hematological and biochemical analysis

Biochemical parameters (total protein, albumin, cholesterol, triglycerides, alanine aminotransferase, aspartate aminotransferase) were measured spectrophotometrically using automated biochemistry analyzer RX Daytona+ (Randox, Crumlin, United Kingdom) and corresponding reagent kits (all Randox, Crumlin, United Kingdom).

Packed cell volume (PCV) was determined using the microhematocrit method [20]. Hemoglobin concentrations were estimated at 550 nm wavelength by hematology analyzer (Beckman Coulter, Brea, California, USA).

Concentrations of serum IgA were measured with commercially available chicken-specific ELISA kit (Biomatik, Wilmington, Delaware, USA). The Assay was performed according to the original manufacturer’s instructions with all samples assayed in duplicate.

### Villi height and crypt depth determination

The jejunum samples were routinely embedded in paraffin, sectioned at the thickness of 4 μm, and stained with hematoxylin and eosin. Measurements were performed on images taken at 40x magnification using a DS-U2 digital camera (Nikon, Amstelveen, Netherlands) and a Nikon Ni/U microscope together with NIS-Elements BR software. A total of 20 intestinal villi and 20 crypts per bird were analyzed. The evaluated morphometric indices included villus height, defined as the distance from the top of the villus to the crypt junction; crypt depth, defined as the depth of the invagination between adjacent villi; and the villus height to crypt depth (VH: CD) ratio.

### Meat quality parameters: analysis of meat color, pH, drip loss and electrical conductivity

Breast muscle pH and temperatures were measured 15 min after sampling and 24 h postmortem using an MA130 Ion Meter (Mettler Toledo, Switzerland). Color of the fresh breast muscle was measured approximately 24 h postmortem and after seven days of storage at 4 °C using a CR 300 colorimeter (Minolta, Osaka, Japan) using illuminant source C and color expressed in terms of CIE values for lightness (L*), redness (a*) and yellowness (b*). The colorimeter was calibrated throughout the study using a standard white ceramic tile. Drip loss was determined by the bag method following previously described protocols [21, 22]. Breast meat samples taken for measuring drip loss were cut 24 h postmortem, weighed, immediately suspended with a thread, put in a plastic bag, sealed, placed to hang freely at 4 °C for 48 h, and re-weighed. Drip loss was expressed as a percentage of weight reduction. Electrical conductivity was measured 24 h post-mortem using LF-Star (Ingenieurbüro Matthäus, Nobitz, Germany).

### SCFA concentration analysis

Concentrations of acetate, propionate, butyrate, iso-butyrate, valerate, isovalerate, and caproate were determined in the cecal contents of one cecum per experimental animal. Frozen cecal samples were thawed on ice, and approximately 1 g of cecal contents was gently squeezed into a pre-weighed 15 ml tube. MilliQ water was added to the weighted samples (1:5, w/v) and vortexed for 3 min. The samples were further homogenized in an ultrasonic bath at a low temperature for 10 min, mixed by vortexing for an additional 5 min, and then centrifuged at 3,200 × g for 5 min at 4 °C. Short-chain fatty acids (SCFAs) were extracted from the supernatants using diethyl ether as described by Adorno et al. (2014), and analyzed with an Agilent J&W DB-WAX capillary column (30 m × 0.25 mm × 0.25 mm) mounted in an Agilent gas chromatograph (6850 N) equipped with a flame ionization detector (FID) and an automatic sample injector (Agilent, USA). Hydrogen was used as the carrier gas. The separation was performed isothermally at 150 °C, with a 250 °C injector and 300 °C detector temperature. ChemStation software (Agilent Technologies, USA) was used for quantification. Concentrations of individual SCFAs were calibrated against crotonate, which was used as the internal standard.

### DNA isolation and sequencing

Each ceca pair was placed into sterile plastic tubes, immediately refrigerated on dry ice (for a maximum of 30 min), and then frozen at -80 °C until further analysis. Frozen cecal samples were thawed on ice, and approximately 1 g of cecal content was gently squeezed into a pre-weighed 15 mL tube. 150 mg of the cecal content was transferred to a bead-beating tube containing lysis buffer from the QIAamp PowerFecal Pro DNA Extraction Kit (Qiagen). The samples were homogenized three times for 30 s at 6000 rpm, each followed by a 15-second cooling period (using a Precellys 24 Homogenizer). The manufacturer’s protocol was followed until DNA was eluted in 75 µL of elution solution. The DNA samples were stored at -20 °C for up to one month before being sent to the sequencing center (Microsynth). Sequencing was performed on the Illumina MiSeq platform using standard Illumina 16 S rRNAV3V4 primers, 2 × 250 cycles, and target depth of 50,000 reads per sample.

### Sequence manipulation and data presentation

The sequences were received demultiplexed with trimmed primer sequences. The samples were merged into a single file, and read allocation to OTUs followed the UPARSE pipeline [23] from USEARCH, with several steps executed using VSEARCH [24]. Stringent parameters were applied for paired read merging and quality filtering when identifying lead OTU sequences, and more relaxed parameters were used during OTU table construction. From the 6.2 million paired reads obtained, 5.4 million sequences were allocated to 665 OTUs. OTUs were further named based on the nearest match from the LTP database [25]. OTU taxonomy was assigned using the Naive Bayesian classifier [26] as implemented in Mothur [27]. Data handling was performed using the R package Phyloseq [28], and visualizations were created with ggplot2.

### Statistical analysis

For statistical analysis GraphPad Prism software, version 8 (GraphPad Software Inc., CA, USA) was used. All CFU data were log transformed before analysis. For performance analysis one pen was considered as one experimental unit. For all other parameters one animal was considered as one experimental unit. All data were tested for normality using Shapiro-Wilk and Kolmogorov-Smirnov test. According to the normality results data were tested for differences in a group with One-way ANOVA with Dunnet’s or Tukey’s multiple comparison test or Kruskal-Wallis test with Dunn’s multiple comparison test. Students’ unpaired t-test or Man-Whitney U-test were used for comparison the of two treatments. Correlation was determined using Spearman r test. Statistical significance is set as *p < 0.05, **p < 0.01, and ***p < 0.001.

Differential expression of OTUs and taxa between test groups was assessed using the R package DESeq2 [29] with a significance threshold of P(adj) < 0.05. Bray-Curtis distances between samples were calculated, and differences among groups were tested using PERMANOVA with the R package pairwise.adonis2 (https://github.com/pmartinezarbizu/pairwiseAdonis/). Differences in alpha diversity between samples were tested using the Wilcoxon rank-sum exact test.

## Results

### Growth performance parameters

**Fig. 2:**
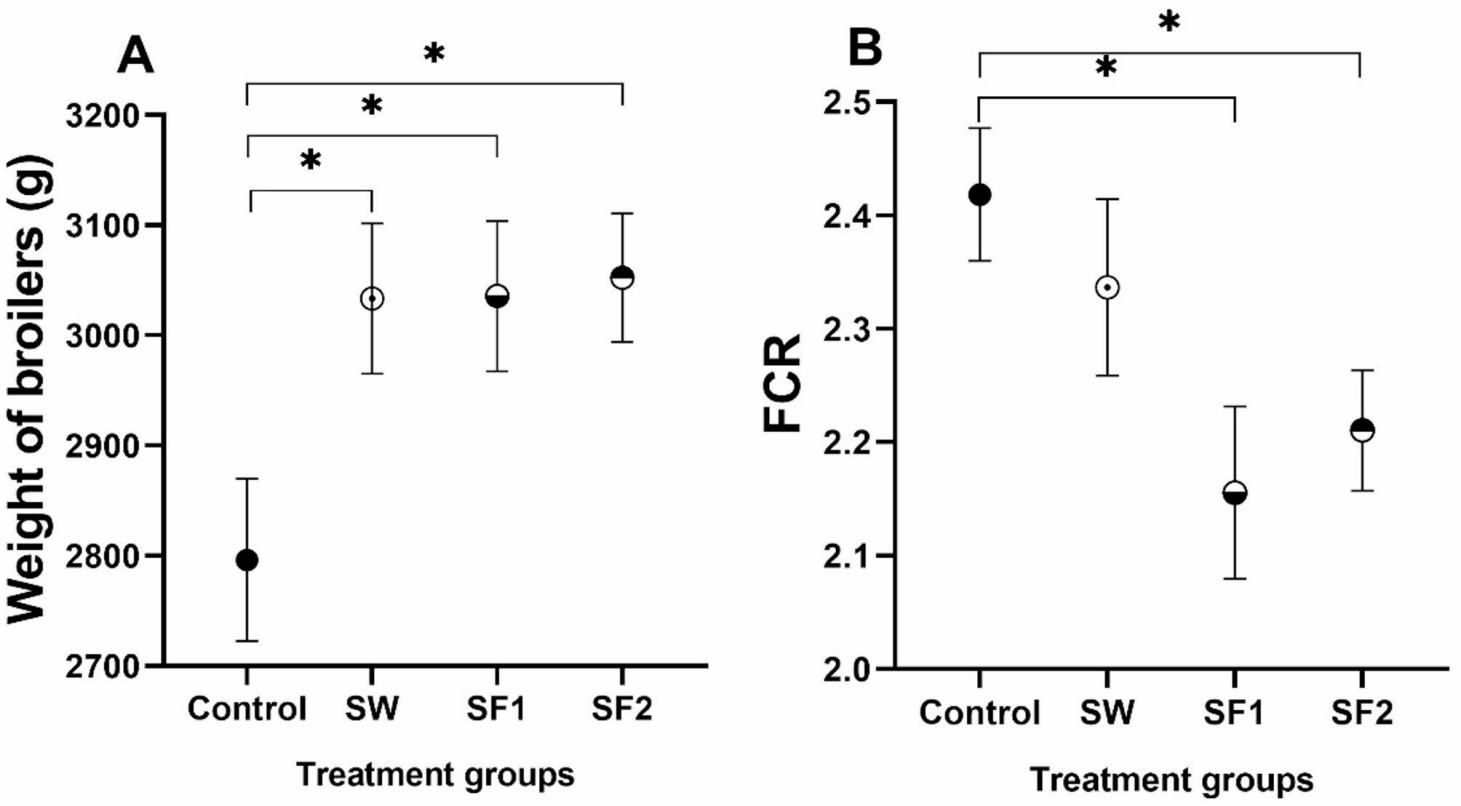
(**A**) Weight of broilers, and (**B**) feed conversion ratio (FCR) at day 43 of untreated (Control) broilers, and broilers treated with *B. subtilis* PS-216 spores in drinking water at the concentration of 10^9^ spores/L (SW), in feed at concentration of 10^6^ spores/kg of feed (SF1), and 10^9^ spores/kg of feed (SF2) presented as mean ± standard error mean. Statistical significance is determined using ANOVA with Dunnett’s multiple comparisons test and presented with *p < 0.05

Early indicators of improved growth performance were observed as early as day 8 in all *B. subtilis* PS-216-treated groups compared to the untreated control (Table S4). However, the most pronounced and statistically significant increases in body weight (BW) occurred at the final weighing (day 43) across all treatment groups (*p* < 0.05). The SW treatment group achieved an average final weight of 3034 g, representing a 7.4% increase over the control group (2842 g). Similarly, the SF1 group reached 3036 g (7.5% improvement), while the SF2 group achieved 3052 g, demonstrating an 8.1% increase compared to the untreated control (Fig. 2A, Table S4).

Feed conversion ratio (FCR) improvements varied among treatment groups. Although the SW group (FCR = 2.3) showed a numerically lower FCR (3% improvement) compared to the untreated control group (FCR = 2.4), this difference did not reach statistical significance (*p* = 0.74). In contrast, both feed-supplemented groups demonstrated significant FCR improvements (*p* < 0.05): SF1 (FCR = 2.2) achieved an 11% improvement, while SF2 (FCR = 2.2) showed a 9% improvement compared to the control (Fig. 2B). The most substantial FCR differences were observed at days 22 and 36 (Table S5). Correlation analysis revealed a significant moderate negative correlation between FCR and spore concentration in feed (*p* = 0.030, *r* = -0.295), indicating enhanced feed efficiency with higher spore concentrations. No significant correlation was detected between final body weight and spore concentration in feed.

### Weight of lymphatic organs and abdominal fat

**Fig. 3:**
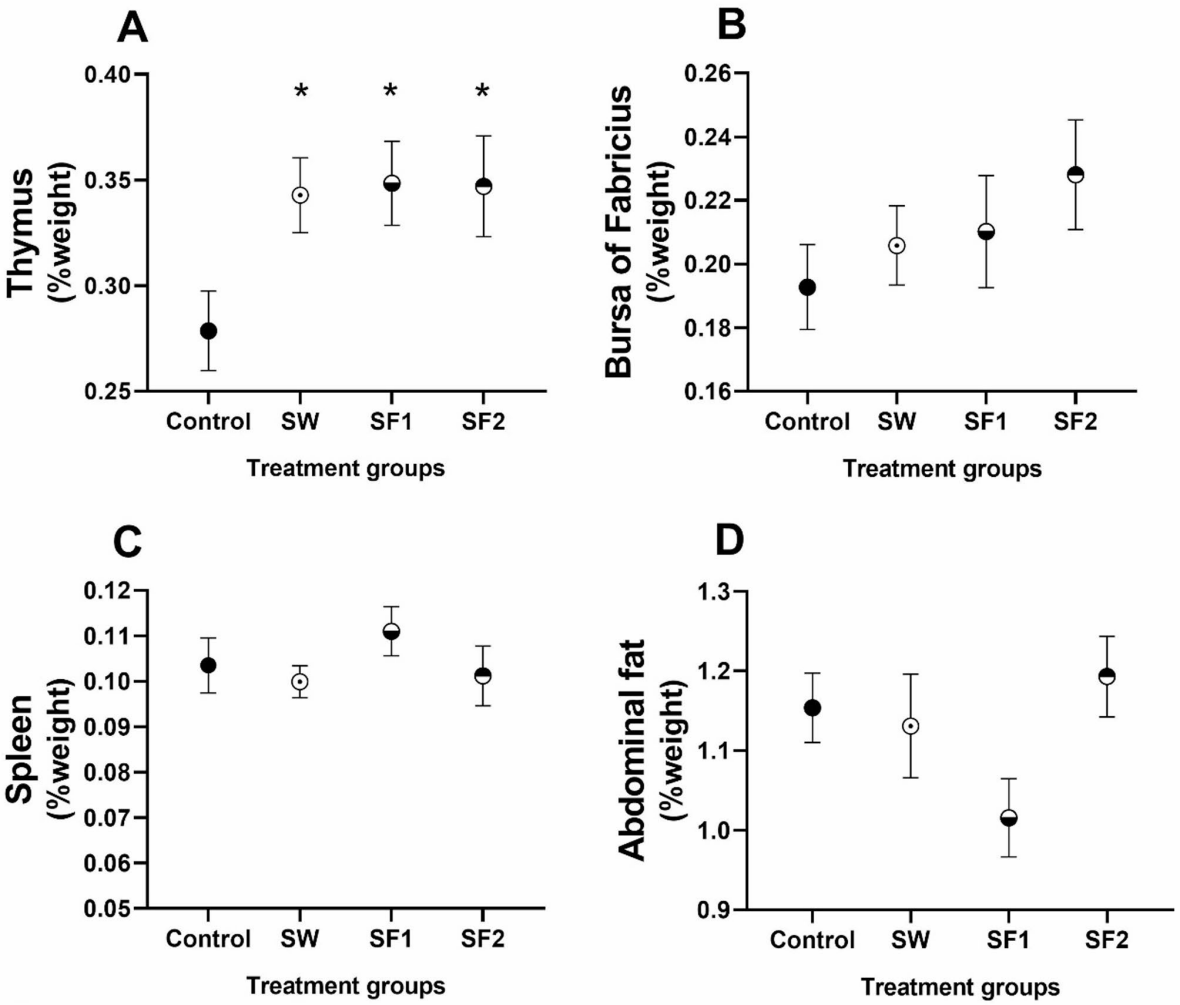
Relative weight (% of organ/carcass) of (**A**) thymus, (**B**) bursa of Fabricius, (**C**) spleen, and (**D**) abdominal fat of broilers untreated (Control) and treated with *B. subtilis* PS-216 spores in drinking water at the concentration of 10^9^ spores/L (SW), in feed at concentration of 10^6^ spores/kg of feed (SF1), and 10^9^ spores/kg of feed (SF2) presented as mean ± standard error mean in second sampling (at day 44). Statistical significance is determined using ANOVA with Dunnett’s multiple comparisons test and presented with *p < 0.05

At the initial sampling point (day 23), no statistically significant differences in lymphatic organ weights were observed among treatment groups (Table S6). However, at the final sampling (day 44), thymus weight, a primary lymphatic organ, was significantly increased by approximately 22% in all treated groups compared to the control (*p* < 0.05; Fig. 3A, Table S7).

Relative thymus weights expressed as percentage of carcass weight were 0.28% (7.9 g) in the control group, 0.34% (10.2 g) in the SW group, 0.35% (10.3 g) in the SF1 group, and 0.35% (10.2 g) in the SF2 group. The bursa of Fabricius weight (Fig. 3B) showed an increase in treatment groups, though this difference was not statistically significant (*p* = 0.29, Table S6). Similarly, spleen weight (Fig. 3C) and abdominal fat deposition (Fig. 3D) did not differ significantly among treatment groups (Tables S6 and S7).

Correlation analysis demonstrated a significant positive relationship between spore concentration in feed and thymus weight, both in absolute terms (*p* = 0.021, *r* = 0.383) and relative to carcass weight (*p* = 0.039, *r* = 0.346).

### Villus height and crypt depth

Spore supplementation did not significantly affect jejunal villus height or crypt depth measurements. Mean villus height was 1203 μm and mean crypt depth was 120 μm across all groups (Table 1). The villus height to crypt depth ratio (VH: CD) was higher in the water-treated group (SW, 11.1) compared to the control (10.4), although this difference did not achieve statistical significance. No significant differences in villus height, crypt depth, or VH: CD ratio were observed among any treatment groups.

**Table 1:** Height of villi (µm), depth of crypts (µm) of the broiler chicken jejunum and their ratio (VH: CD) in untreated control and treated with *B. subtilis* PS-216 spores in drinking water at the concentration of 10^9^ spores/L (SW), in feed at concentration of 10^6^ spores/kg of feed (SF1), and 10^9^ spores/kg of feed (SF2) at second sampling (day 44) with corresponding SEM and statistical significance (p value)

Parameter	Villus height (µm)	Crypt depth (µm)	VH: CD
Control	1205	122	10.4
SW	1219	112	11.1
SF1	1197	127	10.2
SF2	1192	120	10.3
SEM	5.04	2.69	2.69
p value	0.970	0.726	0.669

### Hematological and biochemical parameters of blood samples

All hematological and biochemical parameters remained within normal physiological ranges, with no major alterations observed between the untreated control and feed-supplemented broilers (SF1 and SF2) (Table 2). In the SW group, hemoglobin levels were significantly lower (*p* = 0.026) compared to the untreated control, although values remained within the expected physiological range for broiler chickens.

**Table 2:** Biochemical (cholesterol, triglycerides, AST, ALT, total protein and albumin), hematological parameters (hemoglobin and hematocrit) and serum IgA of broilers untreated control and treated with *B. subtilis* PS-216 spores in drinking water at the concentration of 10^9^ spores/L (SW), in feed at concentration of 10^6^ spores/kg of feed (SF1), and 10^9^ spores/kg of feed (SF2) in second sampling (day 43) with corresponding SEM and statistical significance (*p* value)

Parameter	Cholesterol (mmol/L)	Triglycerides (mmol/L)	AST (U/L)	ALT (U/L)	Total protein (g/L)	Albumin (g/L)	Hemoglobin (g/dl)	Hematocrit (%)	Serum IgA
Control	3.4	0.98	437	1.91	29.6	13.5	8.19	29.2	26,445
SW	3.6	1.05	358	1.31	28.4	13.5	7.77^a^	27.6	27,521
SF1	3.63	1	376	1.68	28.8	13.6	8.2	29.6	26,082
SF2	3.51	0.97	363	3.98	27.6	13.1	7.89	28.4	27,672
SEM	0.04	0.02	15.8	0.52	0.36	0.11	0.09	0.39	340
*p*-value	0.381	0.873	0.47	0.39	0.172	0.537	0.012	0.059	0.863

^a^Statistically significant at *p* < 0.05 as compared to the control determined using One Way ANOVA/Kruskal-Wallis with Dunn’s multiple comparisons test

### Short chain fatty acid profile

**Fig. 4:**
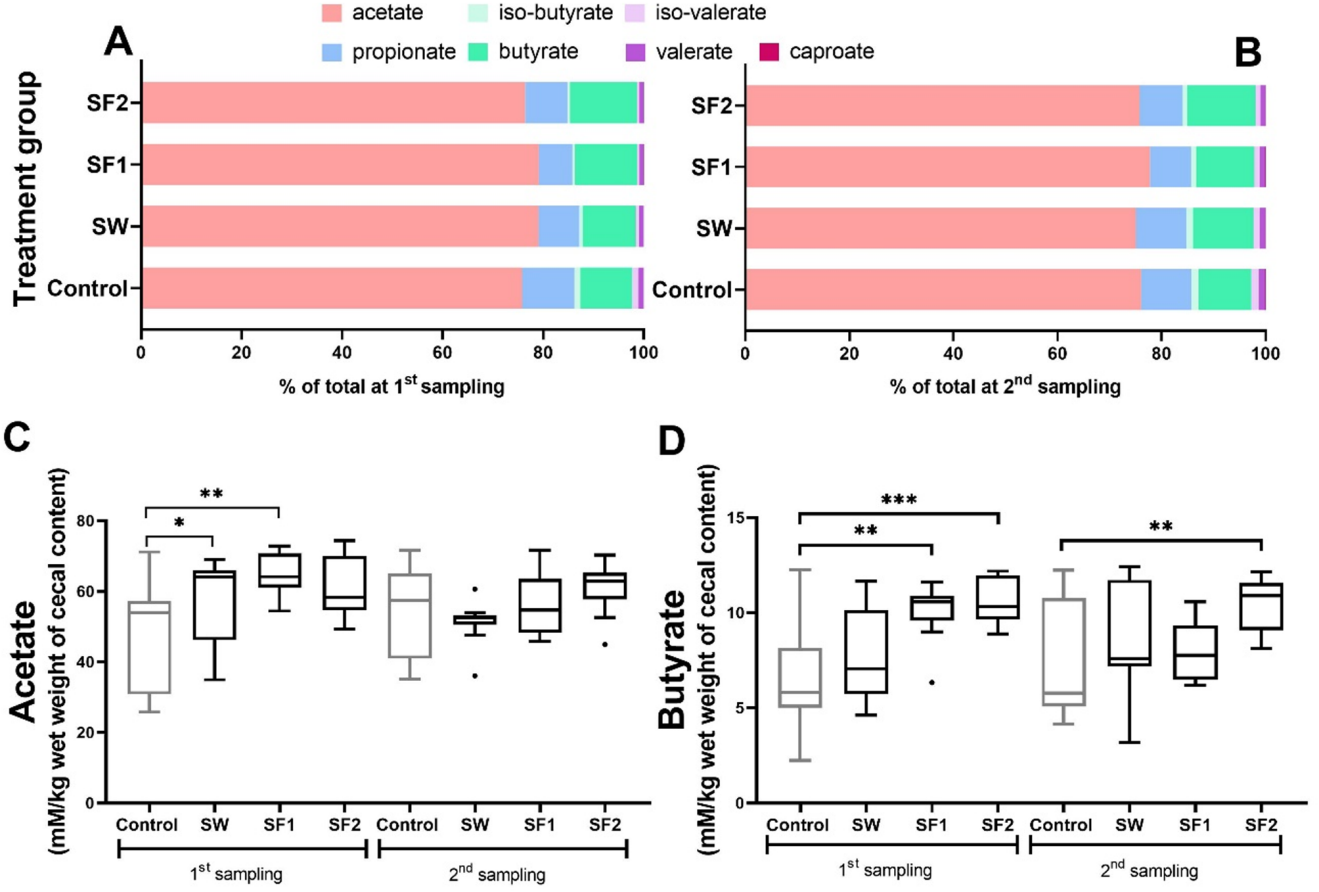
Short chain fatty acid profile in cecum of broilers untreated control and treated with *B. subtilis* PS-216 spores in drinking water at the concentration of 10^9^ spores/L (SW), in feed at concentration of 10^6^ spores/kg of feed (SF1), and 10^9^ spores/kg of feed (SF2) at (**A**) first (day 23) and (**B**) second (at day 44) sampling presented as parts of total SCFAs (%), and more precisely (**C**) acetate and (**D**) butyrate presented as mM of acid per kg wet weight of cecum content. Statistical significance is determined using Kruskal-Wallis with Dunn’s multiple comparisons test and presented with *p < 0.05, **p < 0.01, and ***p < 0.001

*B. subtilis* PS-216 spore supplementation produced more pronounced effects on cecal SCFA profiles at the initial sampling (day 23) compared to the final sampling (day 44). At day 23, treatment groups showed significantly elevated levels of acetate (SW and SF1 groups) and butyrate (SF1 and SF2 groups), alongside reduced concentrations of propionate (SF1), iso-butyrate (SW, SF1, and SF2), and iso-valerate (SF1 and SF2) compared to the untreated control (Fig. 4A, C, and D; Table 3).

**Table 3:** Short chain fatty acids profile (% of total SCFAs) in cecum of broilers untreated control and treated with *B. subtilis* PS-216 spores in drinking water at the concentration of 10^9^ spores/L (SW), in feed at concentration of 10^6^ spores/kg of feed (SF1), and 10^9^ spores/kg of feed (SF2) at first (23 days of age) and second (at 44 days of age) sampling with corresponding SEM and statistical significance (p-value)

	Acetate	Propionate	Iso-butyrate	Butyrate	Iso-valerate	Valerate	Caproate
Parameter at 1st sampling (%)							
Control	75.77	10.44	1.16	10.31	1.23	1.07	0.023
SW	79.05^a^	8.12	0.68^a^	10.56	0.63	0.94	0.012
SF1	79.12^a^	6.69^a^	0.48^a^	12.40^a^	0.45^a^	0.85	0.007
SF2	76.40	8.40	0.53^a^	13.33^a^	0.44^a^	0.90	0.006
SEM	0.76	0.67	0.14	0.63	0.16	0.04	0.003
p-value	0.059	0.055	0.0004	0.007	0.025	0.145	0.787
Parameter at 2nd sampling (%)							
Control	76.06	9.66	1.40	10.11	1.44	1.27	0.058
SW	75.06	9.73	1.24	11.62	1.20	1.11	0.037
SF1	77.77	7.90	0.95	11.17	1.08	1.07	0.056
SF2	75.77	8.27	0.88	13.11^a^	0.93	1.01	0.033
SEM	0.50	0.41	0.10	0.54	0.09	0.05	0.006
p-value	0.327	0.194	0.174	0.012	0.406	0.652	0.309

^a^Statistically significant at *p* < 0.05 as compared to the control determined using Kruskal-Wallis with Dunn’s multiple comparisons test

At the final sampling (day 44), only the SF2 treatment group maintained a significant increase in butyrate concentration compared to the untreated control (Table 3; Fig. 4B, C, and D; *p* < 0.01). SCFA profiles were evaluated both as absolute concentrations (mM/kg wet weight of cecal content) and as proportions relative to total SCFA content. The most substantial changes were observed for acetate and butyrate, with statistically significant differences detected both at the concentration level (Fig. 4C and D) and as percentages of total analyzed SCFAs (Table 3).

### Cecal microbiota diversity and composition

**Fig. 5:**
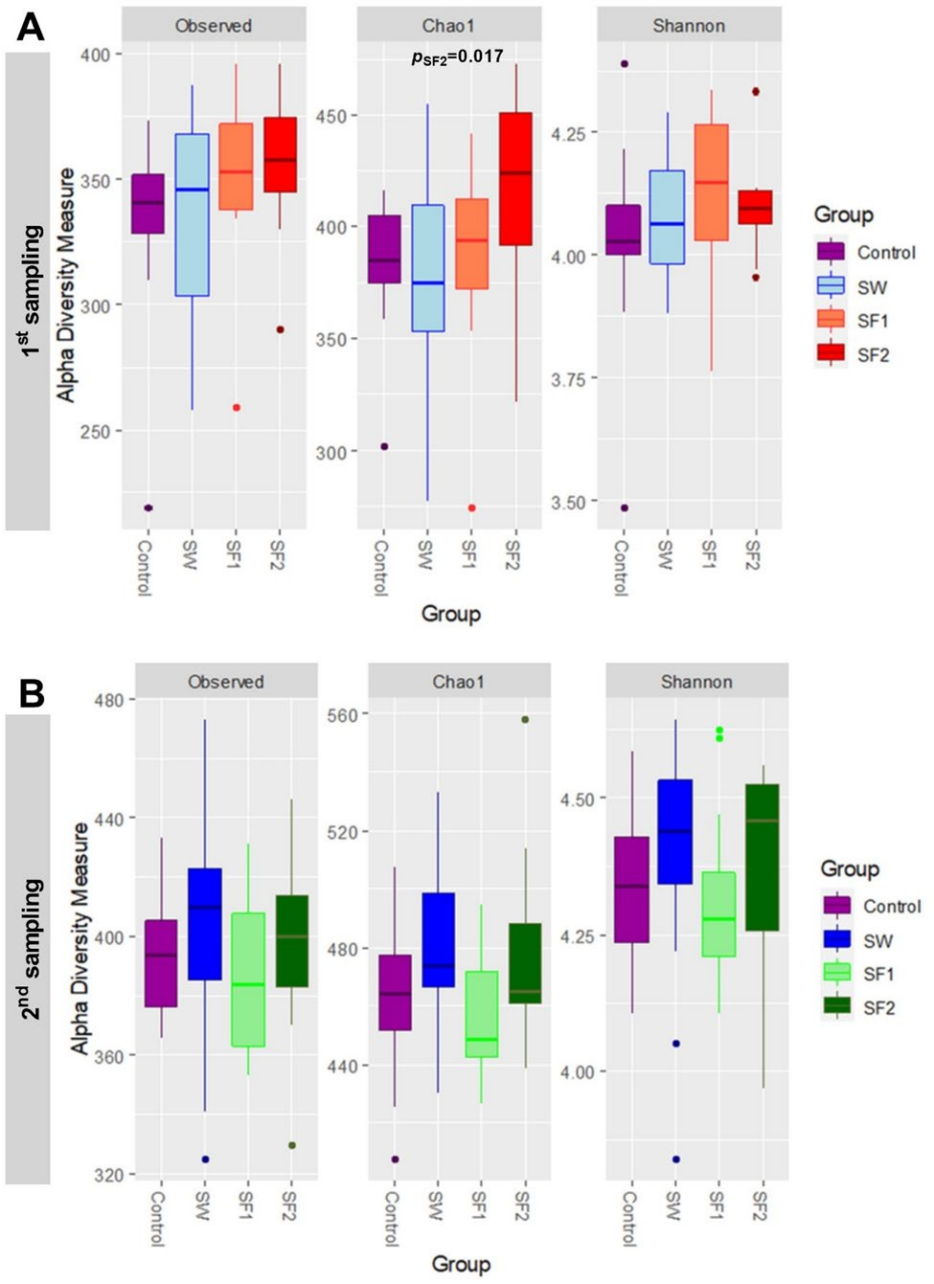
Cecum microbiota alpha diversity measures at (**A**) first sampling (day 23) and (**B**) second sampling (day 44) presented as observed number of OTUs, predicted number of OTUs (Chao1) and Shannon – Weaver diversity index in four experimental groups: broilers untreated Control and treated with *B. subtilis* PS-216 spores in drinking water at the concentration of 10^9^ spores/L (SW), in feed at concentration of 10^6^ spores/kg of feed (SF1), and 10^9^ spores/kg of feed (SF2). Statistical significance is presented as *p* value for Wilcoxon rank sum exact test

**Alpha diversity** of the cecal microbiota was evaluated using three complementary metrics: observed operational taxonomic units (OTUs), the Chao1 richness estimator, and the Shannon diversity index (Fig. 5, Tables S8 and S9). The observed number of OTUs remained consistent across all treatment groups, regardless of administration method (water versus feed) or spore concentration levels.

**Species richness**, as estimated by the Chao1 index, was significantly elevated in the SF2 treatment group (10^9^ spores/kg feed) compared to the untreated control at the initial sampling point (day 23) (*p* = 0.017, Fig. 5A). However, this difference was not maintained at the final sampling (day 44) (*p* = 0.413, Fig. 5B), and no significant differences were observed in the other treatment groups at either time point (Tables S8 and S9). The Shannon diversity index showed no significant differences between any treatment groups and the untreated control across both sampling periods.

**Fig. 6:**
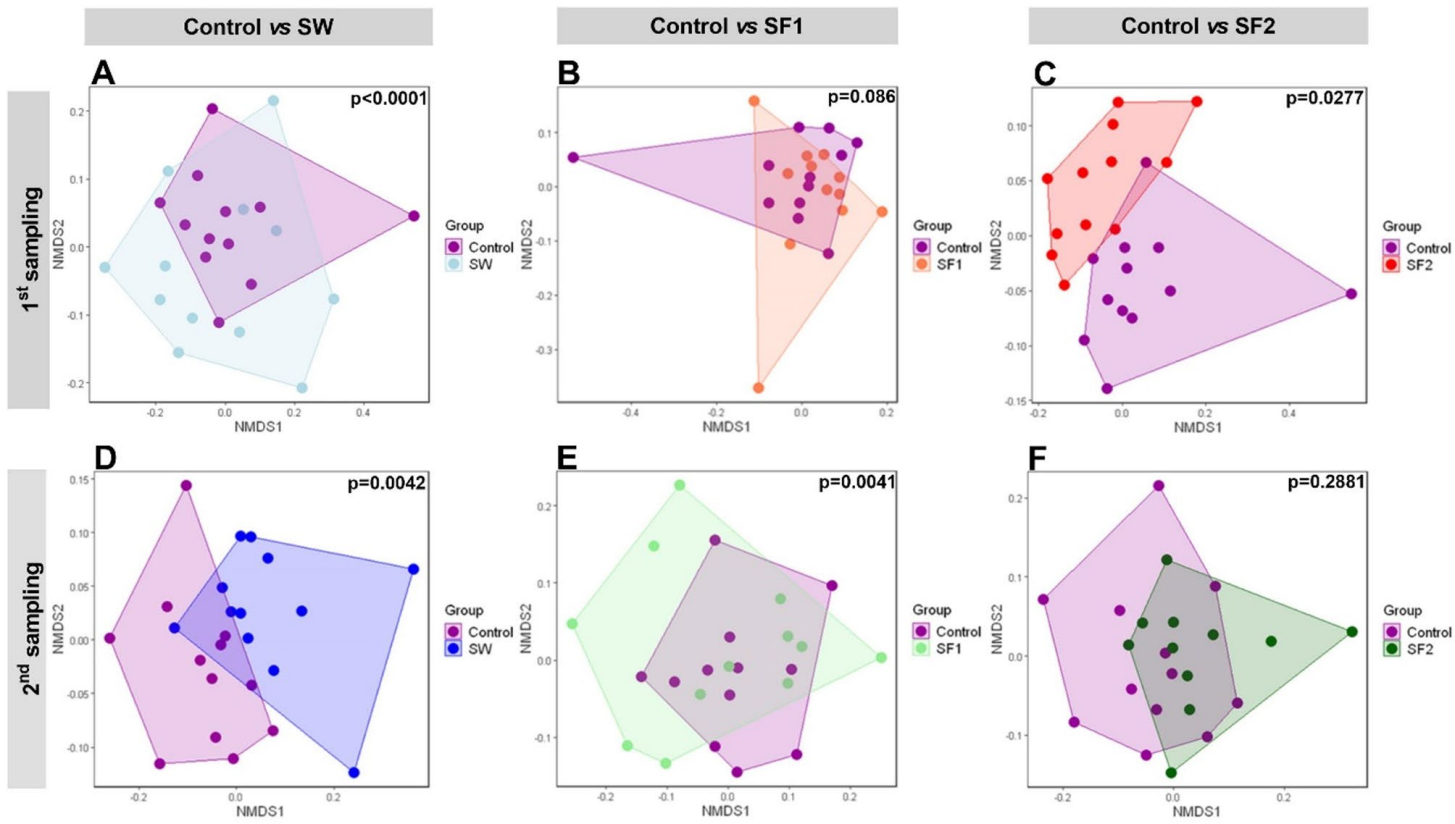
Non-metric multidimensional scaling of Bray-Curtis distances of analyzed microbiota in cecum of broilers at first (day 23) and second (day 44) sampling comparing untreated control with (**A** and **D**) broilers treated with *B. subtilis* PS-216 spores in drinking water at the concentration of 10^9^ spores/L (SW), (**B** and **E**) broilers treated with *B. subtilis* PS-216 spores in feed at concentration of 10^6^ spores/kg of feed (SF1), and (**C** and **F**) broilers treated with *B. subtilis* PS-216 spores in 10^9^ spores/kg of feed (SF2). p-values present statistical significance of comparisons for variance partitioning for Bray-Curtis distances with PairwiseAdonis2 (PERMANOVA)

**Beta diversity** of the cecal microbiota was assessed using non-metric multidimensional scaling (NMDS) ordination of Bray-Curtis dissimilarities. Analysis included comparisons of all treatment groups at both sampling points (Figure S1) and pairwise comparisons of each treatment group against the untreated control (Fig. 6, Tables S10 and S11).

Global comparison of all treatment groups did not reveal significant overall differences in community structure. However, pairwise analyses demonstrated significant compositional shifts at day 23 between the untreated control and both SW (*p* < 0.001) and SF2 (*p* = 0.028) groups, while SF1 showed no significant difference (*p* = 0.086). At day 44, significant differences were observed for SW (*p* = 0.004) and SF1 (*p* = 0.004) groups, but not for SF2 (*p* = 0.288) compared to the untreated control (Table S11, Fig. 6).

The proportion of variance explained by treatment effects (R²) varied by group and sampling time: at day 23, SW, SF1, and SF2 accounted for 9%, 6%, and 7% of the total variance, respectively; at day 44, these values were 9%, 9%, and 5%, respectively (Table S10).

**Fig. 7:**
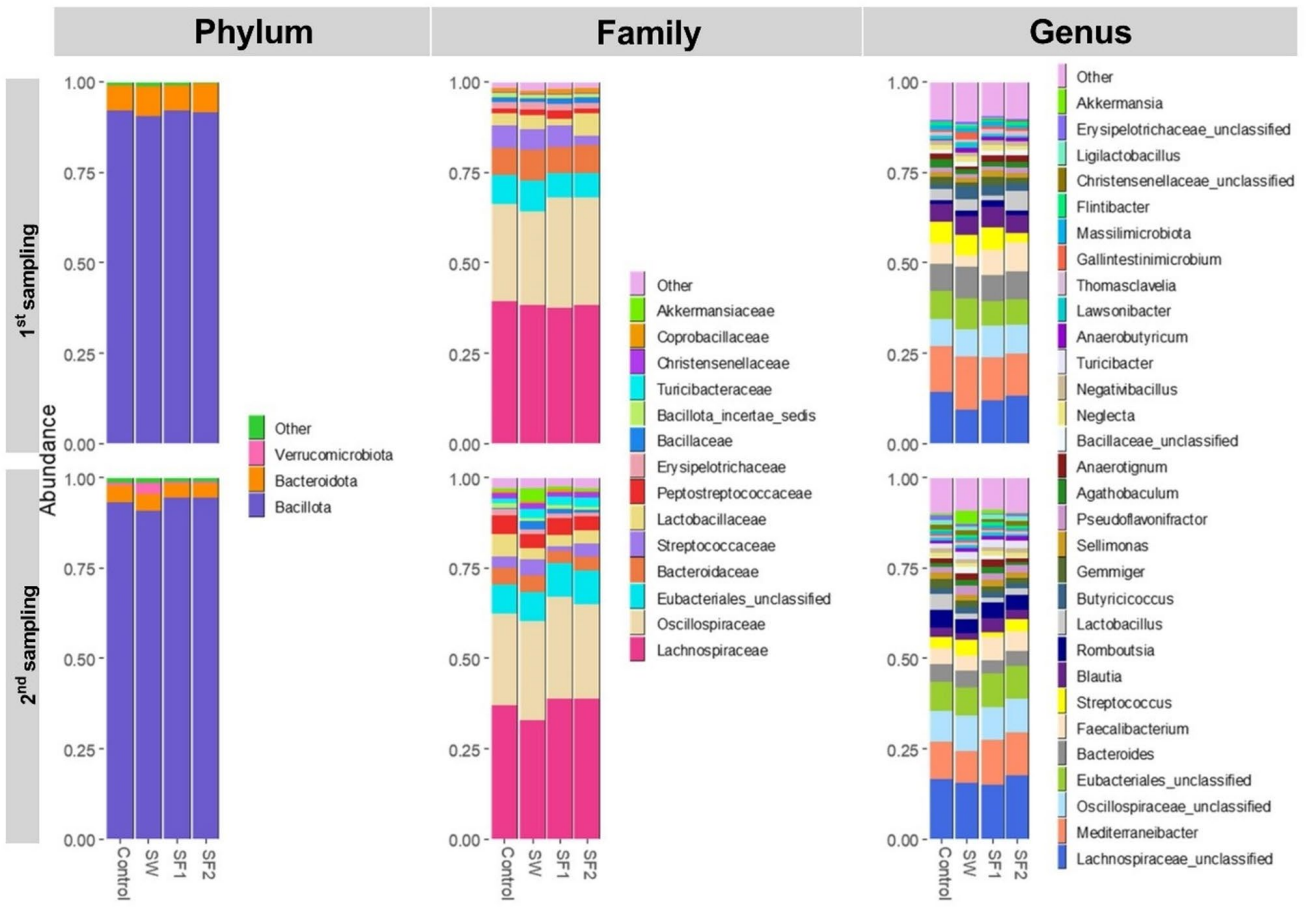
Relative abundance at phylum, family and genus level in cecum microbiota of broilers untreated Control and treated with *B. subtilis* PS-216 spores in drinking water at the concentration of 10^9^ spores/L (SW), in feed at concentration of 10^6^ spores/kg of feed (SF1), and 10^9^ spores/kg of feed (SF2) at first sampling at day 23 and second sampling at day 44

#### Phylum-Level changes

At the phylum level, significant reductions in *Mycoplasmatota* were observed in SW (Log₂FC = -4.07, *p* < 0.0001) and SF2 (Log₂FC = -3.01, *p* = 0.0034) treatment groups at the final sampling (day 44). The SF1 treatment group showed no significant reduction in this phylum (Fig. 7, Table S12, Figure S2). No significant phylum-level differences were detected at the initial sampling (day 23).

**Family-Level changes** At day 23, the SF2 treatment group exhibited significant increases in three bacterial families compared to the untreated control: *Turicibacteraceae* (Log₂FC = 3.80, *p* = 0.0194), *Peptococcaceae* (Log₂FC = 1.76, *p* = 0.0407), and *Christensenellaceae* (Log₂FC = 1.34, *p* = 0.0453) (Fig. 7, Table S12, Figure S3). No significant family-level differences were observed for SW and SF1 groups at this time point. At day 44, all three treatment groups demonstrated significant compositional changes compared to the control. The most pronounced increase was observed in unclassified *Selenomonadales* across all treatment groups (Log₂FC > 20, *p* < 0.0001). Additional increases were noted in *Clostridiaceae_1* (SF2 and SW groups), *Staphylococcaceae* and *Brevibacteriaceae* (SF1 and SW groups), and *Bacillaceae*, *Carnobacteriaceae*, *Dermabacteraceae*, and *Corynebacteriaceae* (SW group only) (Table S12, Figure S4). Conversely, *Anaeroplasmataceae* decreased significantly in SF2 (Log₂FC = -3.02, *p* = 0.0063) and SW (Log₂FC = -4.14, *p* < 0.0001) groups.

#### Genus-Level changes

At day 23, genus-level alterations included increased *Anaerobutyricum* (Log₂FC = 1.54, *p* = 0.0278) in the SW group, and elevated *Turicibacter* (Log₂FC = 3.84, *p* = 0.0179) with reduced *Salibaculum* (Log₂FC = -2.68, *p* = 0.0104) in the SF2 group (Fig. 7, Table S12, Figure S5). At day 44, more extensive genus-level modifications were observed. *Clostridium* sensu stricto increased in both SF2 and SW groups, while SW treatment alone showed increases in *Mammaliicoccus*, unclassified *Bacillaceae*, *Jeotgalicoccus*, *Brevibacterium*, *Brachybacterium*, *Staphylococcus*, *Anaerotruncus*, *Corynebacterium*, and *Pseudoflavonifractor*. Decreases were noted in *Anaeroplasma* (SF2 and SW groups) and unclassified *Erysipelotrichaceae* (SF1 group) (Table S12, Figure S6).

**Individual OTU Analysis** Analysis at the individual OTU level revealed the most extensive differences between the SW treatment group and untreated control, with 10 differentially abundant OTUs at day 23 and 18 at day 44 (Table S13, Figures S7 and S8). Two OTUs showed consistent patterns across treatment groups: OTU233 (*Guopingia tenuis* from *Christensenellaceae*) increased in all treatment groups at day 23 (Log₂FC > 3, *p* < 0.05), while OTU273 (*Anaerosinus glycerini* from *Sporomusaceae*) increased across all groups at day 44 (Log₂FC > 20, *p* < 0.0001).

### Meat quality

**Fig. 8:**
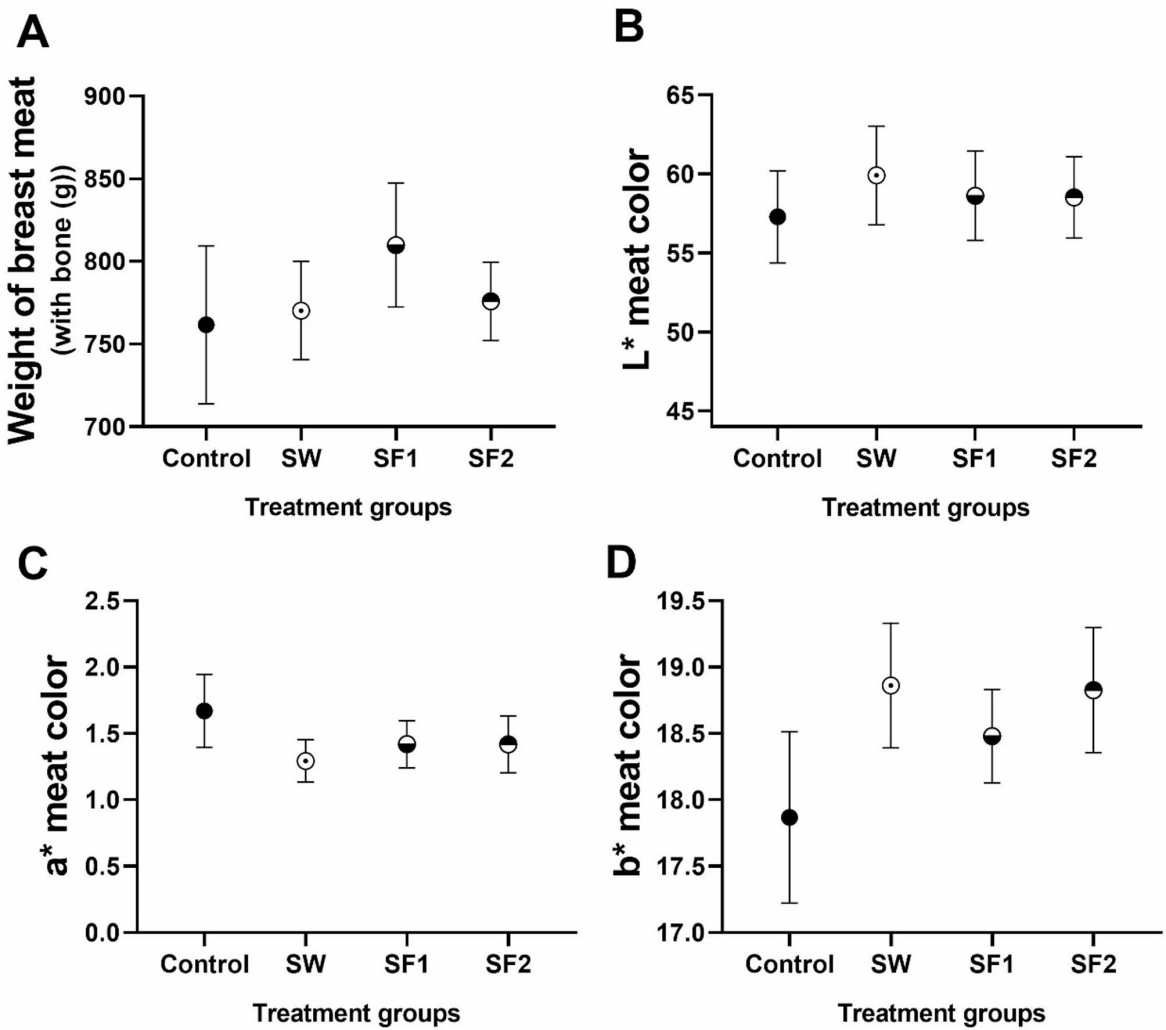
(**A**) Weight of breast meat with bone (g), (**B**) lightness (L*), (**C**) redness (a*) and (**D**) yellowness (b*) of meat samples of control group and groups treated with *B. subtilis* PS-216 spores in drinking water at the concentration of 10^9^ spores/L (SW), in feed at concentration of 10^6^ spores/kg of feed (SF1), and 10^9^ spores/kg of feed (SF2) presented as mean ± standard error mean

Meat quality evaluation was conducted on breast muscle samples from four animals per pen (*n* = 12 per treatment group), assessing weight, color, pH, electrical conductivity, and drip loss parameters. No statistically significant differences were observed in breast meat weight or overall color parameters between treated chickens and the untreated control (Figs. 8A-D).

**Fig. 9:**
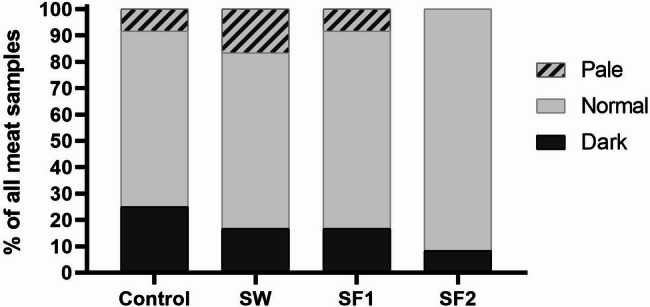
Proportion (%) of meat samples categorized into dark (L*<56), normal (56 ≤ L*≤62) and pale (L*>62) in regards to broiler treatment type: untreated (Control), treated with *B. subtilis* PS-216 spores in drinking water at the concentration of 10^9^ spores/L (SW), in feed at concentration of 10^6^ spores/kg of feed (SF1), and 10^9^ spores/kg of feed (SF2)

**Color analysis** revealed numerical but non-significant trends in specific parameters. Redness (a*) values were higher in the control group (1.7) compared to treated groups: SW (1.3), SF1 (1.4), and SF2 (1.4), though statistical significance was not achieved. Conversely, yellowness (b*) values showed the opposite pattern, with the control group exhibiting lower values (17.9) compared to SW (18.9), SF1 (18.5), and SF2 (18.8), again without statistical significance. All treatment groups demonstrated average meat lightness (L*) values within the normal range (56 ≤ L* ≤ 62) as defined by Lee et al. (2022). Although average lightness values did not differ significantly among groups (Table S7), spore-treated samples showed higher values, prompting further analysis of meat quality categorization. Detailed analysis of individual samples revealed distinct distribution patterns across meat quality categories (Fig. 9).

In the control group, 25% of samples (*n* = 3/12) were classified as dark (L* < 56), 67% (*n* = 8/12) as normal (56 ≤ L* ≤ 62), and 8% (*n* = 1/12) as pale (L* >62). The SW treatment group showed 17% dark samples (*n* = 2/12), 67% normal (*n* = 8/12), and 17% pale (*n* = 2/12). The SF1 group demonstrated a similar pattern with 17% dark (*n* = 2/12), 75% normal (*n* = 9/12), and 8% pale (*n* = 1/12). Most notably, the SF2 treatment group (10^9^ spores/kg feed) exhibited the most favorable distribution, with only 8% dark samples (*n* = 1/12) and 92% normal lightness (*n* = 11/12), with no pale samples observed.

**Fig. 10:**
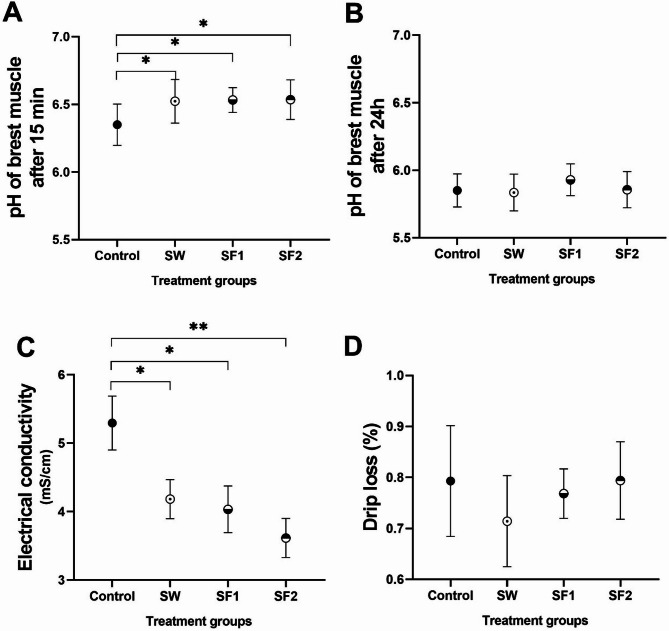
pH values of meat samples (**A**) 15 min and (**B**) 24 h after slaughter and (**C**) electrical conductivity and (**D**) drip loss of meat samples of control group and groups treated with *B. subtilis* PS-216 spores in drinking water at the concentration of 10^9^ spores/L (SW), in feed at concentration of 10^6^ spores/kg of feed (SF1), and 10^9^ spores/kg of feed (SF2) presented as mean ± standard deviation. Statistical significance is determined using ANOVA with Tukey’s multiple comparisons test and presented with *p < 0.05 and **p < 0.01

**Breast meat pH** was evaluated at two time points post-slaughter: 15 min and 24 h (Fig. 10A and B). At 15 min post-slaughter, the control group exhibited significantly lower pH values (6.3) compared to all treatment groups (*p* < 0.05): SW (pH = 6.5), SF1 (pH = 6.5), and SF2 (pH = 6.5). At 24 h post-slaughter, all samples achieved pH values within the normal range (5.7–6.1), with no significant differences among groups.

**Electrical conductivity** measurements revealed significant improvements in meat quality parameters for all treatment groups compared to the control. The untreated control breast meat showed electrical conductivity of 5.3 mS/cm, which was significantly higher than all treatment groups: 21% higher than SW (4.2 mS/cm, *p* < 0.05), 24% higher than SF1 (4.0 mS/cm, *p* < 0.05), and 32% higher than SF2 (3.6 mS/cm, *p* < 0.05) (Fig. 10C). Correlation analysis demonstrated a significant negative relationship between electrical conductivity and spore concentration in feed (*p* = 0.002, *r* = -0.504), indicating improved meat quality with higher supplementation levels.

**Breast meat drip loss** remained consistent across all treatment groups and the untreated control, ranging from 0.71% to 0.79% on average, with no statistically significant differences observed among groups (Fig. 10D).

## Discussion

This study investigated the effects of *B. subtilis* PS-216 spore supplementation on broiler performance, immune parameters, cecal microbiota, SCFA composition, and meat quality. Spores were administered via drinking water (10^9^ spores/L; SW group; consistent with our previous study [13]) or feed at two concentrations (10^6^ spores/kg, SF1; 10^9^ spores/kg, SF2), with feed supplementation representing a more practical delivery method.

*B. subtilis* PS-216 supplementation significantly improved performance parameters, with increased body weight and reduced FCR compared to controls. Weight gains were evident early, from day 8, with the most pronounced differences at final weighing, consistent with previous *Bacillus*-based probiotic studies [30–33]. Feed supplementation (SF1 and SF2) demonstrated superior FCR improvements compared to water treatment, with a moderate negative correlation (*r* = -0.295) between FCR and spore concentration. Positive changes in performance parameters are likely a consequence of an overall beneficial effect of probiotics, including immunomodulatory effect, modulation of microbiota, changes in SCFA profile, improvements in intestinal histomorphology, and pathogen control [7, 8, 10].

The significant increase in thymus weight across all treatment groups, particularly the positive correlation with spore concentration (*r* = 0.383), indicates enhanced immune status. This immunomodulatory effect aligns with previous studies showing increased lymphatic organ weights following *Bacillus* supplementation [34–37], though responses vary by strain specificity. The immunomodulatory properties of *Bacillus* species have been attributed to various compounds, such as exopolysaccharides (EPS) [38, 39] and surfactants [40]. Even spores, which are dormant life forms of *Bacillu*s have been shown to contribute to immunomodulation [9].

The overall health and immune status of an animal is greatly influenced by the state of the gut, including composition of the microbiome, and SCFA profiles. Here, cecal SCFA profiles demonstrated beneficial shifts, particularly increased butyrate and acetate concentrations alongside decreased iso-butyrate and iso-valerate levels. These changes indicate improved gut health through enhanced carbohydrate fermentation and reduced protein fermentation. This creates a more favorable environment for beneficial members of the microbiota while limiting pathogenic bacteria [41–44]. Butyrate elevation is particularly important given its anti-inflammatory properties, pathogen control capabilities, and systemic health benefits [41, 43, 45, 46]. Feed supplementation was superior to that by water (SF2 vs. SW), as the largest differences in butyrate and iso-valerate occurred in feed treated groups, indicating that the type of treatment also affects the gut microbiota activity.

This work shows that the addition of *B. subtilis* PS-216 in both feed and water modulates the composition of the microbiota, revealing treatment-influenced modulation effects. Alpha diversity increased only in the SF2 group at day 23, while beta diversity analysis demonstrated compositional shifts at both sampling points across all treatment groups. Notable changes included significant reductions in *Mycoplasmatota* phylum, specifically *Anaeroplasma*, which typically increases under stress conditions [47, 48]. Conversely, beneficial butyrate-producing genera (*Selenomonadales*, *Clostridium* sensu stricto) increased across treatment groups [49, 50]. The water treatment group showed additional increases in *Pseudoflavonifractor* and *Anaerotruncus*, genera associated with IL-22 and TNF-α production, suggesting enhanced tissue protection and immune regulation [51, 52]. These microbiota changes support an indirect mechanism by which PS-216 enhances butyrate production through modulation of cecal bacterial communities.

The composition and functionality of the gut microbiota is essential for the wellbeing of chickens. A reduction in microbiota richness and diversity, coupled with the overgrowth of harmful microbes, can occur during stress and may lead to disease and reduced flock productivity [53, 54]. Therefore, maintaining a stable microbiota enriched for specific beneficial taxonomic groups is essential for promoting broiler health, production, meat quality and safety. However, meat quality indicators comprising color, pH, drip loss, and electrical conductivity may vary with chicken genetics and depend on consumer preferences, which differ by region [55, 56]. In this study, the meat quality assessment revealed subtle but meaningful improvements. While most parameters showed no significant differences, the SF2 group demonstrated optimal meat lightness distribution, with 92% of samples falling within normal ranges compared to 67% in controls. Electrical conductivity was significantly reduced across all treatment groups, indicating improved water-holding capacity and meat quality [57]. Although drip loss was similar across treatment groups, the relatively small sample size (12 animals per treatment) may have limited the ability to detect meaningful differences.

Feed supplementation at 10^9^ spores/kg (SF2) showed the most comprehensive benefits, affecting body weight, FCR, thymus weight, SCFA profiles, and microbiota composition. Even the lower concentration (SF1, 10^6^ spores/kg) improved key performance parameters, suggesting dose-dependent effects while maintaining practical applicability.

## Conclusion

*B. subtilis* PS-216 is a well-known laboratory strain isolated from the bank soil of the river Sava in Slovenia, extensively studied for bacterial communication and inter- and intra-species interactions. This study demonstrates that *B. subtilis* PS-216 functions as an effective probiotic in broiler chickens, conferring multifaceted benefits across performance, immune function, gut health, and meat quality parameters. Feed supplementation at 10^9^ spores/kg of feed provided optimal results, significantly improving body weight, feed conversion ratio, and thymus weight while beneficially modulating cecal microbiota composition and SCFA profiles.

The strain’s ability to enhance butyrate production through microbiota modulation, reduce stress-associated bacterial populations (*Mycoplasmatota*), and improve meat quality indicators positions *B. subtilis* PS-216 as a promising alternative to antibiotic growth promoters in sustainable poultry production. The dose-dependent responses and administration route effects provide practical guidance for commercial implementation.

In this study no *Campylobacter* specific sequences were detected in the gut microbiota. Future research should thus evaluate PS-216’s pathogen control efficacy in *Campylobacter*-colonized broilers and include comparative strain analysis to elucidate the specific mechanisms underlying probiotic effectiveness in spore-forming bacteria. These investigations will advance our understanding of *Bacillus*-based probiotics and support their strategic application in antibiotic-free poultry production systems.

## Supplementary Information

Below is the link to the electronic supplementary material.

Supplementary Material 1

## Data Availability

The sequence data has been submitted to the NCBI BioProject database under ID PRJNA1249408, link https://dataview.ncbi.nlm.nih.gov/object/PRJNA1249408?reviewer=2kbsrkvmmbdvoov2ui9l2s6f4h. All data with statistical analysis is available at FigShare 10.6084/m9.figshare.28623206, link https://figshare.com/articles/dataset/_b_Probiotic_effect_of_b_b_i_B_subtilis_i_b_b_PS-216_in_broiler_chickens_modulation_of_weight_feed_conversion_short_chain_fatty_acids_microbiota_and_meat_quality_b_/28623206 and with the corresponding author upon request.
