# Supplementary material for: Probiotic effect of B. subtilis PS-216 in broiler chickens: modulation of weight, feed conversion, short chain fatty acids, microbiota and meat quality
Source: Anim Microbiome. 2025 Nov 19;7:121. doi: 10.1186/s42523-025-00489-7 (PMC12628548; doi:10.1186/s42523-025-00489-7)
Supplement: Supplementary file 1 — Supplementary Material 1 [file 42523_2025_489_MOESM1_ESM.docx]

**Probiotic effect of *B. subtilis* PS-216 in broiler chickens: modulation of weight, feed conversion, SCFA, microbiota and meat quality**

**Supplementary Information**

Table S1. Composition and calculated nutrient content of the experimental diets fed to broiler chickens during the starter (1-15 d) and finisher (16-43 d) period.

|  | Starter | Finisher |
| --- | --- | --- |
| Composition of experimental diets^1^, % |  |  |
| Maize | 42.65 | 42.76 |
| Wheat | 15.00 | 20.00 |
| Soya meal | 35.48 | 29.35 |
| Molasses | 0.02 | - |
| Plant oil | 2.87 | 4.05 |
| L-lysine-HCl | 0.28 | 0.29 |
| DL-methionine | 0.21 | 0.22 |
| L-threonine | 0.14 | 0.11 |
| Monocalcium phosphate | 0.90 | 0.74 |
| Limestone | 1.26 | 1.05 |
| Salt | 0.22 | 0.23 |
| Mineral-vitamin supplement^2,3^ | 0.50 | 0.50 |
| Calculated energy and nutrient content |  |  |
| Metabolizable energy, MJ/kg | 12.40 | 13.10 |
| Crude protein, % | 21.00 | 19.00 |
| Lysine, % | 1.30 | 1.15 |
| Methionine, % | 0.50 | 0.48 |
| Calcium, % | 1.00 | 0.90 |
| Available phosphorus, % | 0.57 | 0.52 |
| Sodium, % | 0.18 | 0.18 |

^1^All diets contain coccidiostat Maxiban® G160 (Elanco Products Co., Hook, Hampshire, UK).

^2^Calculated to meet the mineral and vitamin requirements for Ross 308 broiler starter diets and provided per kilogram of the diet: Cu 15 mg, Fe 50 mg, Mn 100 mg, Se 0.3 mg, Zn 80 mg, vitamin A 13.500 IU, vitamin D_3_ 5000 IU, vitamin E 80 IU, vitamin K 4.08 mg, thiamine (B_1_) 4.02 mg, riboflavin (B_2_) 12.0 mg, niacin (B_3_) 70.4 mg, pyridoxine (B_6_) 5.05 mg, biotin 0.20 mg, folic acid 1.97 mg, vitamin B_12_ 0.03 mg.

^3^Calculated to meet the mineral and vitamin requirements for Ross 308 broiler finisher diets and provided per kilogram of the diet: Cu 15 mg, Fe 40 mg, Mn 100 mg, Se 0.3 mg, Zn 74 mg, vitamin A 10.000 IU, vitamin D_3_ 5000 IU, vitamin E 55 IU, vitamin K 3,2 mg, thiamine (B_1_) 3.0 mg, riboflavin (B_2_) 12.0 mg, niacin (B_3_) 70.4 mg, pyridoxine (B_6_) 3.05 mg, biotin 0.20 mg, folic acid 1.8 mg, vitamin B_12_ 0.0016 mg.

Table S2. Proximate composition and concentration of minerals of the starter experimental diets.

| Component | Dietary treatments^1^ | | |  |
| --- | --- | --- | --- | --- |
|  | Control/SW | SF1 | SF2 | |
| Dry matter, g/kg | 881.95 | 882.73 | 881.92 | |
| Crude protein, g/kg | 205.42 | 206.30 | 207.34 | |
| Crude fat, g/kg | 46.12 | 46.95 | 46.89 | |
| Crude ash, g/kg | 53.08 | 51.02 | 53.50 | |
| Crude fiber, g/kg | 37.70 | 36.87 | 38.12 | |
| Nitrogen free extract, g/kg | 539.63 | 541.59 | 536.07 | |
| K, g/kg | 11.85 | 12.04 | 11.90 | |
| Ca, g/kg | 8.08 | 8.47 | 8.61 | |
| P, g/kg | 5.88 | 5.52 | 5.34 | |
| Mg, g/kg | 2.13 | 2.19 | 2.13 | |
| Na, g/kg | 1.30 | 1.29 | 1.64 | |
| Fe, mg/kg | 191.75 | 166.63 | 170.13 | |
| Zn, mg/kg | 169.69 | 178.13 | 161.25 | |
| Mn, mg/kg | 146.31 | 160.31 | 160.81 | |
| Cu, mg/kg | 22.68 | 22.50 | 22.49 | |

^1^All values are means of two analyses per measured sample.

Table S3. Proximate composition and concentration of minerals of the finisher experimental diets.

| Component | Dietary treatments^1^ | | |  |
| --- | --- | --- | --- | --- |
|  | Control/SW | SF1 | SF2 | |
| Dry matter, g/kg | 881.95 | 882.73 | 881.92 | |
| Crude protein, g/kg | 190.41 | 191.01 | 187.45 | |
| Crude fat, g/kg | 63.40 | 59.86 | 62.64 | |
| Crude ash, g/kg | 53.68 | 55.15 | 54.55 | |
| Crude fiber, g/kg | 38.05 | 38.30 | 38.13 | |
| Nitrogen free extract, g/kg | 543.14 | 545.60 | 548.19 | |
| K, g/kg | 10.44 | 10.85 | 10.27 | |
| Ca, g/kg | 8.94 | 8.91 | 9.05 | |
| P, g/kg | 5.34 | 5.43 | 5.37 | |
| Mg, g/kg | 2.10 | 2.12 | 2.10 | |
| Na, g/kg | 1.63 | 1.54 | 1.70 | |
| Fe, mg/kg | 173.88 | 166.44 | 176.88 | |
| Zn, mg/kg | 155.94 | 144.38 | 147.19 | |
| Mn, mg/kg | 196.88 | 164.00 | 153.38 | |
| Cu, mg/kg | 18.15 | 19.68 | 17.98 | |

*^1^All values are means of two analyses per measured sample.*

Table S4. Body weight of broiler chickens untreated control and treated with *B. subtilis* PS-216 spores in drinking water at the concentration of 10^9^ spores/L (SW), in feed at concentration of 10^6^ spores/kg of feed (SF1), and 10^9^ spores/kg of feed (SF2) at days 1, 8, 15, 22, 29, 36 and 42 with corresponding SEM and statistical significance (p value).

|  | Body weight average (g) | | | |  |  |
| --- | --- | --- | --- | --- | --- | --- |
| **Day** | **Control** | **SW** | **SF1** | **SF2** | **SEM** | ***p* - value** |
| D1 | 43 | 43 | 43 | 43 | 0.1 | 0.971 |
| D8 | 147 | 153 | 162 | 163 | 3.7 | 0.003 |
| D15 | 411 | 421 | 432 | 437 | 5.9 | 0.205 |
| D22 | 845 | 864 | 886 | 880 | 9.3 | 0.448 |
| D29 | 1415 | 1418 | 1473 | 1492 | 19.3 | 0.396 |
| D36 | 2124 | 2232 | 2252 | 2279 | 33.8 | 0.212 |
| D43 | 2824 | 3034 | 3036 | 3052 | 54.3 | 0.026 |

Table S5. Feed conversion ratio (FCR) of broiler chickens untreated control and treated with *B. subtilis* PS-216 spores in drinking water at the concentration of 10^9^ spores/L (SW), in feed at concentration of 10^6^ spores/kg of feed (SF1), and 10^9^ spores/kg of feed (SF2) at days 8, 15, 22, 29, 36 and 42 with corresponding SEM and statistical significance (p value).

|  | FCR | | | |  |  |
| --- | --- | --- | --- | --- | --- | --- |
| **Day** | **Control** | **SW** | **SF1** | **SF2** | **SEM** | **p value** |
| D8 | 2.22 | 2.22 | 2.05 | 1.93 | 0.07 | 0.367 |
| D15 | 2.26 | 2.18 | 2.07 | 2.09 | 0.53 | 0.552 |
| D22 | 2.23 | 2.23 | 1.62 | 2.18 | 0.15 | 0.008 |
| D29 | 2.78 | 2.80 | 2.50 | 2.53 | 0.08 | 0.228 |
| D36 | 2.49 | 2.22 | 2.38 | 2.19 | 0.07 | 0.144 |
| D43 | 2.52 | 2.37 | 2.35 | 2.34 | 0.04 | 0.625 |

Table S6. Weight (g) and relative weight (%) of thymus, bursa of Fabricius and spleen of broiler chickens untreated control and treated with *B. subtilis* PS-216 spores in drinking water at the concentration of 10^9^ spores/L (SW), in feed at concentration of 10^6^ spores/kg of feed (SF1), and 10^9^ spores/kg of feed (SF2) at first sampling at 23 days of age with corresponding SEM and statistical significance (p value).

|  | First sampling | | | | | |
| --- | --- | --- | --- | --- | --- | --- |
| **Parameter** | **Control** | **SW** | **SF1** | **SF2** | **SEM** | **p value** |
| Thymus (g) | 2.94 | 3.62 | 3.29 | 3.38 | 0.14 | 0.300 |
| Thymus (%) | 0.32 | 0.35 | 0.32 | 0.33 | 0.01 | 0.695 |
| Bursa of Fabricius (g) | 2.61 | 2.92 | 2.56 | 2.81 | 0.08 | 0.435 |
| Bursa of Fabricius (%) | 0.28 | 0.29 | 0.25 | 0.28 | 0.01 | 0.598 |
| Spleen (g) | 0.66 | 0.81 | 0.64 | 0.66 | 0.04 | 0.188 |
| Spleen (%) | 0.07 | 0.08 | 0.06 | 0.07 | 0.00 | 0.243 |

Table S7. Weight (g) and relative weight (%) of thymus, bursa of Fabricius, spleen, abdominal fat, breast muscle meat with bone, breast muscle meat of left side, pH, drip loss, electrical conductivity and color (L*, a*, b*) of broiler chickens untreated control and treated with *B. subtilis* PS-216 spores in drinking water at the concentration of 10^9^ spores/L (SW), in feed at concentration of 10^6^ spores/kg of feed (SF1), and 10^9^ spores/kg of feed (SF2) at second sampling at 44 days with corresponding SEM and statistical significance (p value).

|  | Second sampling | | | | | |
| --- | --- | --- | --- | --- | --- | --- |
| **Parameter** | **Control** | **SW** | **SF1** | **SF2** | **SEM** | **p value** |
| Thymus (g) | 7.94 | 10.23 | 10.25 | 10.25 | 0.58 | 0.009 |
| Thymus (%) | 0.28 | 0.34 | 0.35 | 0.35 | 0.02 | 0.05 |
| Bursa of Fabricius (g) | 5.48 | 6.14 | 6.25 | 6.81 | 0.27 | 0.293 |
| Bursa of Fabricius (%) | 0.19 | 0.21 | 0.21 | 0.23 | 0.01 | 0.444 |
| Spleen (g) | 2.96 | 3.00 | 3.32 | 3.00 | 0.08 | 0.505 |
| Spleen (%) | 0.103 | 0.100 | 0.111 | 0.101 | 0.00 | 0.494 |
| Abdominal fat (g) | 33.33 | 33.94 | 30.26 | 35.56 | 1.11 | 0.31 |
| Abdominal fat (%) | 1.15 | 1.13 | 1.03 | 1.19 | 0.04 | 0.113 |
| Breast muscle meat  with bone (g) | 761.7 | 770.3 | 810.0 | 776.0 | 10.6 | 0.790 |
| Breast muscle  meat with bone (%) | 26.28 | 25.63 | 27.12 | 26.10 | 0.31 | 0.35 |
| Breast muscle, left (g) | 328 | 327 | 346 | 332 | 4.40 | 0.867 |
| Breast muscle, left (%) | 11.26 | 10.88 | 11.58 | 11.16 | 0.14 | 0.439 |
| pH 15 min | 6.35 | 6.52 | 6.53 | 6.53 | 0.05 | 0.005 |
| pH 24h | 5.85 | 5.84 | 5.93 | 5.86 | 0.02 | 0.29 |
| Drip loss (%) | 0.79 | 0.71 | 0.77 | 0.79 | 0.02 | 0.896 |
| Electrical conductivity (mS/cm) | 5.30 | 4.18 | 4.03 | 3.62 | 0.36 | 0.006 |
| Color L* (lightness) | 57 | 60 | 59 | 59 | 0.54 | 0.186 |
| Color a* (redness) | 1.67 | 1.29 | 1.42 | 1.42 | 0.08 | 0.636 |
| Color b* (yellowness) | 17.9 | 18.9 | 18.5 | 18.8 | 0.23 | 0.467 |

Table S8: Alpha diversity measures presented as median values for observed number of OTUs, predicted number of OTUs (Chao1) and Shannon–Weaver diversity index of groups of broiler chickens untreated control and treated with *B. subtilis* PS-216 spores in drinking water at the concentration of 10^9^ spores/L (SW), in feed at concentration of 10^6^ spores/kg of feed (SF1), and 10^9^ spores/kg of feed (SF2).

|  | 1^st^ sampling | | | |
| --- | --- | --- | --- | --- |
| Median | **Control** | **SW** | **SF1** | **SF2** |
| Shannon | 4.03 | 4.06 | 4.15 | 4.09 |
| Chao1 | 384 | 375 | 394 | 424 |
| Observed | 341 | 346 | 353 | 358 |
|  | 2^nd^ sampling | | | |
| Shannon | 4.34 | 4.44 | 4.28 | 4.46 |
| Chao1 | 464 | 474 | 449 | 465 |
| Observed | 394 | 410 | 384 | 400 |

Table S9: Statistical significance presented as P values for Wilcoxon rank sum exact test, of alpha diversity measures of test groups of broiler chickens treated with *B. subtilis* PS-216 spores in drinking water at the concentration of 10^9^ spores/L (SW), in feed at concentration of 10^6^ spores/kg of feed (SF1), and 10^9^ spores/kg of feed (SF2) compared to untreated control.

|  | 1^st^ sampling | | |
| --- | --- | --- | --- |
| P | **SW** | **SF1** | **SF2** |
| Shannon | 0.713 | 0.128 | 0.319 |
| Chao1 | 0.755 | 0.799 | 0.017 |
| Observed | 0.795 | 0.141 | 0.073 |
| P | 2^nd^ sampling | | |
| Shannon | 0.319 | 0.713 | 0.449 |
| Chao1 | 0.114 | 0.478 | 0.413 |
| Observed | 0.291 | 0.514 | 0.442 |


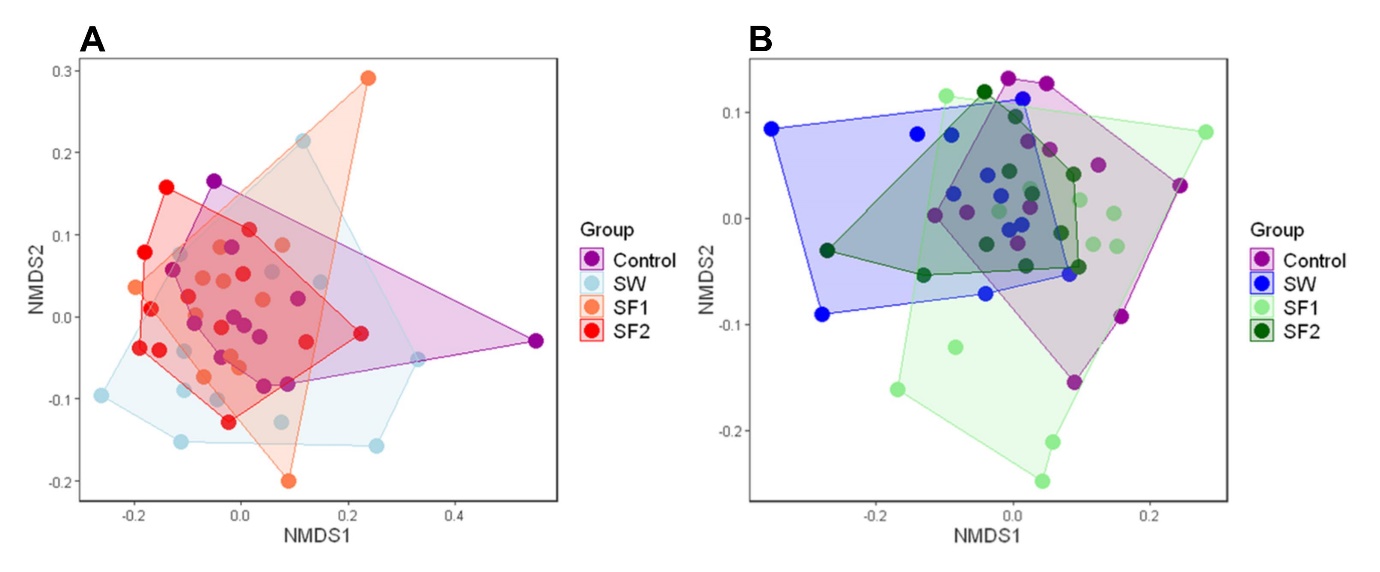


Figure S1. Non-metric multidimensional scaling of Bray-Curtis distances of analyzed microbiota in cecum of broiler chickens untreated control and treated with *B. subtilis* PS-216 spores in drinking water at the concentration of 10^9^ spores/L (SW), in feed at concentration of 10^6^ spores/kg of feed (SF1), and 10^9^ spores/kg of feed (SF2) at **(A)** first (23 days) and **(B)** second (at 44 days) sampling.

Table S10. Variance partitioning for Bray-Curtis distances with PairwiseAdonis2 (PERMANOVA). Test groups (SW, SF1, SF2) compared to control group.

| 1^st^ sampling at 23 days | | | |
| --- | --- | --- | --- |
|  | **SW** | **SF1** | **SF2** |
| Pr | 0.0007 | 0.086 | 0.028 |
| R^2^ | 0.090 | 0.060 | 0.072 |
| Pseudo-F | 2.18 | 1.40 | 1.72 |
| 2^nd^ sampling at 44 days | | | |
| Pr | 0.004 | 0.004 | 0.288 |
| R^2^ | 0.089 | 0.091 | 0.049 |
| Pseudo-F | 2.14 | 2.20 | 1.12 |

Table S11. Statistical significance (p-values) of all comparisons for variance partitioning for Bray-Curtis distances with PairwiseAdonis2 (PERMANOVA).

| 1^st^ sampling | | | | |
| --- | --- | --- | --- | --- |
| *p*-value | **Control** | **SW** | **SF1** | **SF2** |
| Control | - |  |  |  |
| SW | 7.00E-04 | - |  |  |
| SF1 | 0.0862 | 0.0052 | - |  |
| SF2 | 0.0277 | 4.00E-04 | 0.0042 | - |
| 2^nd^ sampling | | | | |
| Control | - |  |  |  |
| SW | 0.0042 | - |  |  |
| SF1 | 0.0041 | 0.0039 | - |  |
| SF2 | 0.2881 | 0.0061 | 0.0384 | - |

Table S12. Statistically significant differences in cecum microbiota at phylum, family and genus level of broiler chickens treated with *B. subtilis* PS-216 spores in drinking water at the concentration of 10^9^ spores/L (SW), in feed at concentration of 10^6^ spores/kg of feed (SF1), and 10^9^ spores/kg of feed (SF2) compared to the untreated control, at first (23 days) and second (at 44 days) sampling presented as Log2 fold change (Log_2_FC) and the adjusted p value.

| **Phylum** | | |
| --- | --- | --- |
|  | Log_2_FC | P_adjusted_ |
| 2^nd^ sampling: SF2 vs Control | | |
| *Mycoplasmatota* | -3.01 | 0.0034 |
| 2^nd^ sampling: SW vs Control | | |
| *Mycoplasmatota* | -4.07 | 0.0000 |
| **Family** | | |
|  | Log_2_FC | P_adjusted_ |
| 1^st^ sampling: SF2 vs Control | | |
| *Turicibacteraceae* | 3.80 | 0.0194 |
| *Peptococcaceae* | 1.76 | 0.0407 |
| *Christensenellaceae* | 1.34 | 0.0453 |
| 2^nd^ sampling: SF2 vs Control | | |
| *Selenomonadales* unclassified | 23.20 | 0.0000 |
| *Clostridiaceae_1* | 5.22 | 0.0004 |
| *Anaeroplasmataceae* | -3.02 | 0.0063 |
| 2^nd^ sampling: SF1 vs Control | | |
| *Selenomonadales* unclassified | 21.63 | 0.0000 |
| *Staphylococcaceae* | 1.97 | 0.0419 |
| *Brevibacteriaceae* | 2.34 | 0.0419 |
| 2^nd^ sampling: SW vs Control | | |
| *Selenomonadales* unclassified | 22.65 | 0.0000 |
| *Anaeroplasmataceae* | -4.14 | 0.0000 |
| *Staphylococcaceae* | 2.84 | 0.0002 |
| *Bacillaceae* | 2.73 | 0.0003 |
| *Clostridiaceae_1* | 4.25 | 0.0032 |
| *Brevibacteriaceae* | 2.66 | 0.0043 |
| *Dermabacteraceae* | 2.76 | 0.0048 |
| *Carnobacteriaceae* | 3.14 | 0.0073 |
| *Corynebacteriaceae* | 2.12 | 0.0123 |
| **Genus** | | |
|  | Log_2_FC | P_adjusted_ |
| 1^st^ sampling: SF2 vs Control | | |
| *Solibaculum* | -2.68 | 0.0104 |
| *Turicibacter* | 3.84 | 0.0179 |
| 1^st^ sampling: SW vs Control | | |
| *Anaerobutyricum* | 1.54 | 0.0278 |
| 2^nd^ sampling: SF2 vs Control | | |
| *Selenomonadales* unclassified | 23.08 | 0.0000 |
| *Clostridium sensu stricto* | 5.34 | 0.0006 |
| *Anaeroplasma* | -2.97 | 0.0161 |
| 2^nd^ sampling: SF1 vs Control | | |
| *Selenomonadales* unclassified | 21.63 | 0.0000 |
| *Erysipelotrichaceae* unclassified | -1.00 | 0.0143 |
| 2^nd^ sampling: SW vs Control | | |
| *Selenomonadales* unclassified | 22.76 | 0.0000 |
| *Anaeroplasma* | -3.95 | 0.0001 |
| *Mammaliicoccus* | 2.75 | 0.0020 |
| *Bacillaceae* unclassified | 2.52 | 0.0020 |
| *Jeotgalicoccus* | 2.96 | 0.0020 |
| *Clostridium sensu stricto* | 4.38 | 0.0035 |
| *Brevibacterium* | 2.63 | 0.0092 |
| *Brachybacterium* | 2.72 | 0.0107 |
| *Staphylococcus* | 2.68 | 0.0124 |
| *Anaerotruncus* | 2.18 | 0.0124 |
| *Jeotgalibaca* | 3.12 | 0.0128 |
| *Corynebacterium* | 2.07 | 0.0243 |
| *Pseudoflavonifractor* | 1.29 | 0.0305 |

Figure S2. Statistically significant differences in cecum microbiota at Phylum level of broiler chickens treated with *B. subtilis* PS-216 spores in drinking water at the concentration of 10^9^ spores/L (SW), in feed at concentration of 10^6^ spores/kg of feed (SF1), and 10^9^ spores/kg of feed (SF2) compared to the untreated control, at second (at 44 days) sampling showing abundance of *Mycoplasmatota*.

Figure S3. Statistically significant differences in cecum microbiota at Family level of broiler chickens treated with *B. subtilis* PS-216 spores in drinking water at the concentration of 10^9^ spores/L (SW), in feed at concentration of 10^6^ spores/kg of feed (SF1), and 10^9^ spores/kg of feed (SF2) compared to the untreated control, at first sampling (23 days) showing abundance of *Turibacteraceae, Peptococcaceae and Christensenellaceae.*


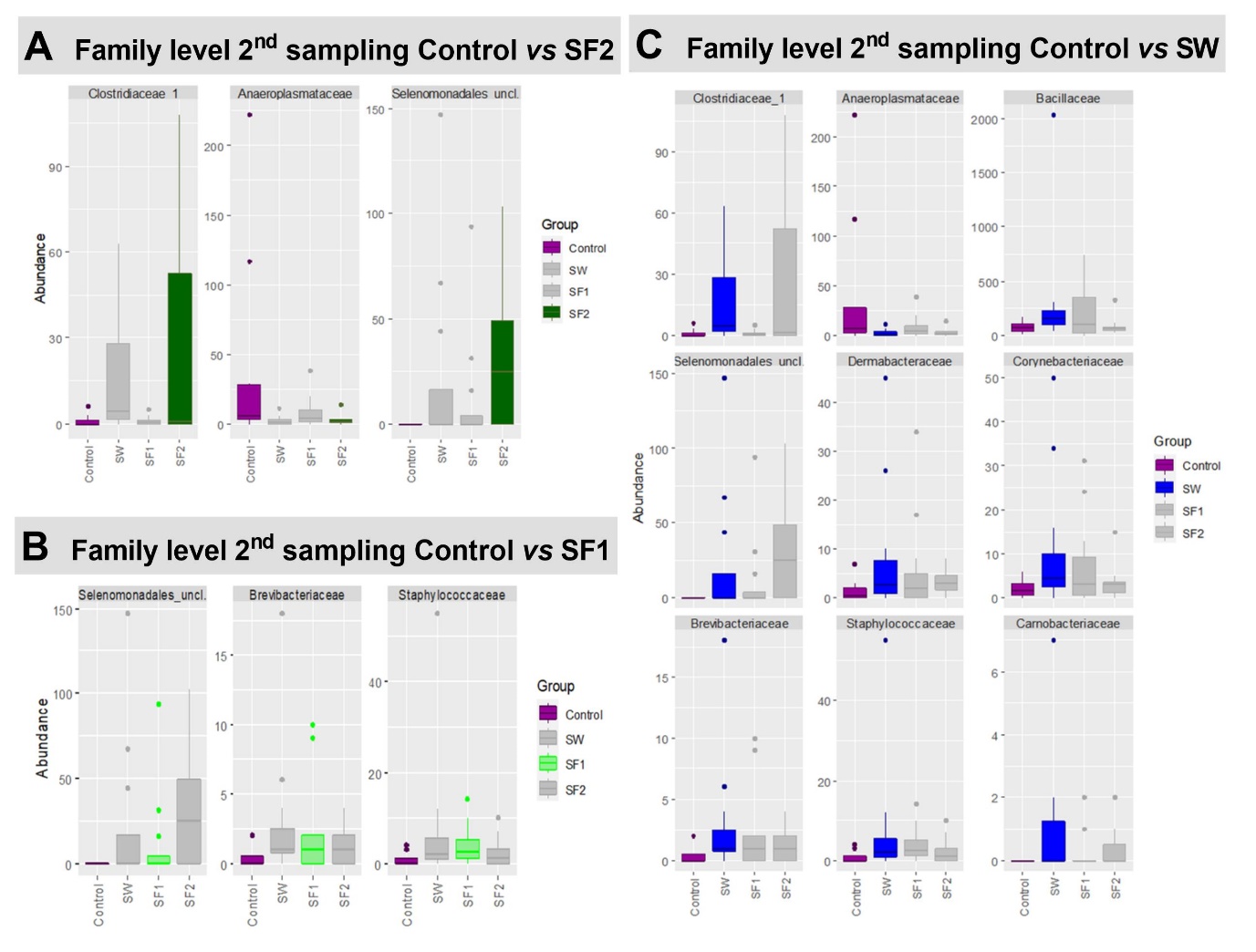


Figure S4. Abundance of cecum microbiota members at Family level with statistically significant differences in abundance in treatment groups **(A)** SF2 (spores in feed at concentration 10^9^ spores/kg of feed), **(B)** SF1 (spores in feed at concentration of 10^6^ spores/kg of feed) and **(C)** SW (*B. subtilis* PS-216 spores drinking water at the concentration of 10^9^ spores/L) compared to the untreated control, at second sampling (44 days).


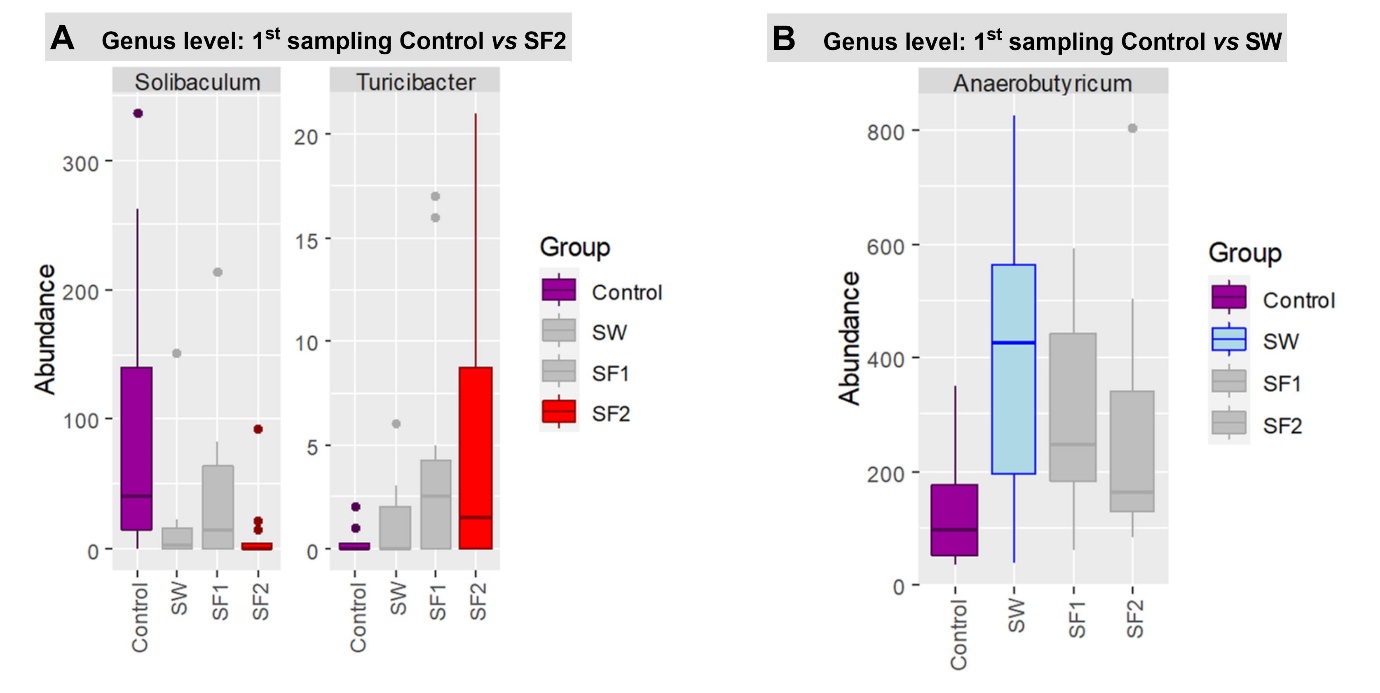


Figure S5. Abundance of cecum microbiota members at Genus level with statistically significant differences in abundance in treatment groups **(A)** SF2 (spores in feed at concentration 10^9^ spores/kg of feed), **(B)** SF1 (spores in feed at concentration of 10^6^ spores/kg of feed) and **(C)** SW (*B. subtilis* PS-216 spores drinking water at the concentration of 10^9^ spores/L) compared to the untreated control, at first sampling (23 days).


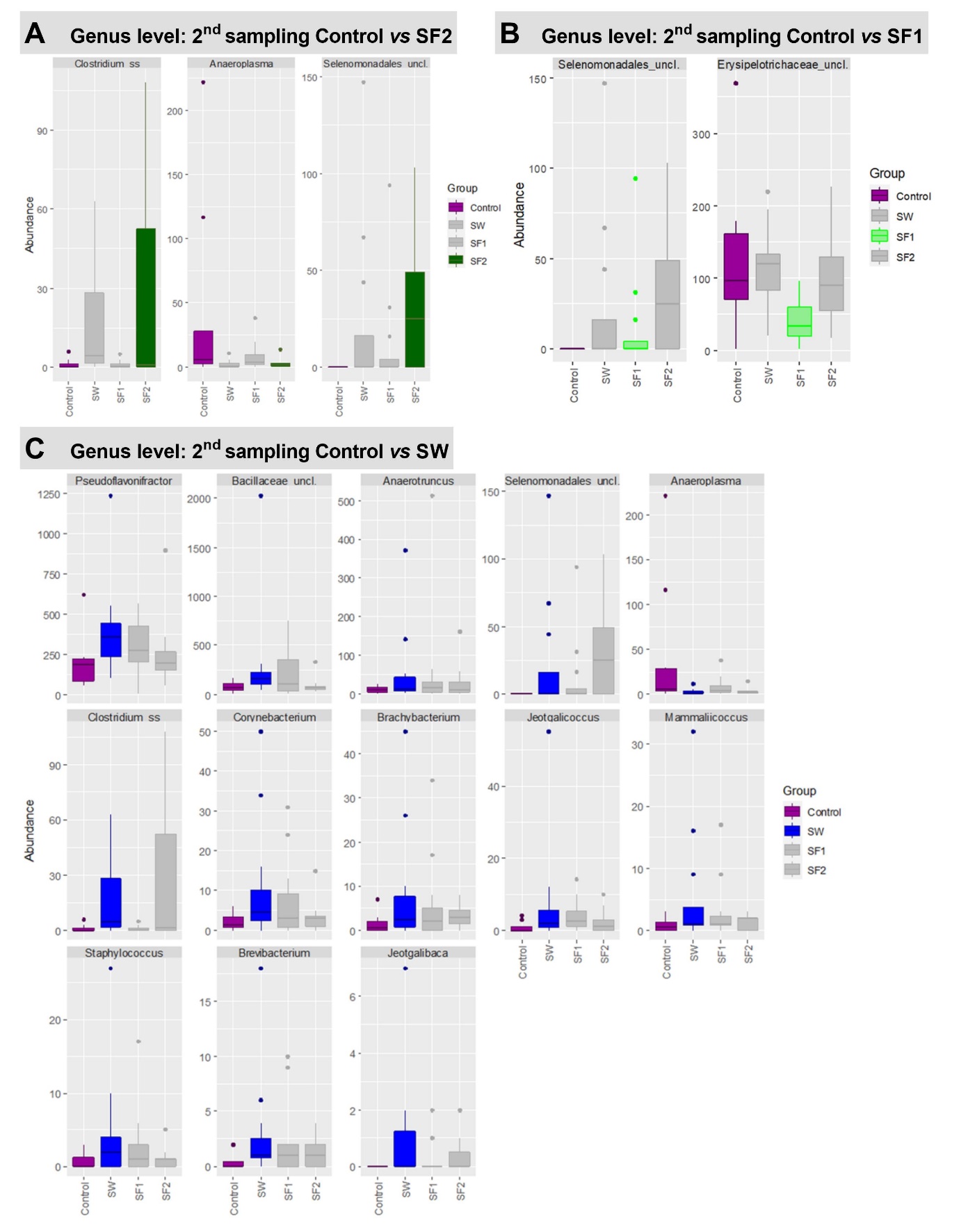


Figure S6. Abundance of cecum microbiota members at Genus level with statistically significant differences in abundance in treatment groups **(A)** SF2 (spores in feed at concentration 10^9^ spores/kg of feed), **(B)** SF1 (spores in feed at concentration of 10^6^ spores/kg of feed) and **(C)** SW (*B. subtilis* PS-216 spores drinking water at the concentration of 10^9^ spores/L) compared to the untreated control, at second sampling (44 days).

Table S13. Statistically significant differences in cecum microbiota at individual OUT level of broiler chickens treated with *B. subtilis* PS-216 spores in drinking water at the concentration of 10^9^ spores/L (SW), in feed at concentration of 10^6^ spores/kg of feed (SF1), and 10^9^ spores/kg of feed (SF2) compared to the untreated control, at first (23 days) and second (at 44 days) sampling presented as Log2 fold change (L2FC) and the adjusted p value.

|  | **SW** | | **SF1** | | **SF2** | |
| --- | --- | --- | --- | --- | --- | --- |
|  | L_2_FC | p_adj_ | L_2_FC | p_adj_ | L_2_FC | p_adj_ |
| **1^st^ sampling day 23** | | | | | | |
| OTU_165_Faecalicoccus_acidiformans_Erysipelotrichaceae_94_4 | 2.1 | 0.003 | 1.8 | 0.004 |  |  |
| OTU_227_Massiliimalia_massiliensis_Oscillospiraceae_95_3 | 2.1 | 0.019 | - |  |  |  |
| OTU_233_Guopingia_tenuis_Christensenellaceae_84_7 | 4.8 | 0.001 | 3.7 | 0.030 | 4.0 | 0.005 |
| OTU_331_Lawsonibacter_celer_Oscillospiraceae_97_5 | 3.0 | 0.003 |  |  |  |  |
| OTU_367_Clostridium_fessum_Clostridiaceae_96 | 2.2 | 0.049 |  |  | 2.8 | 0.004 |
| OTU_38_Anaerobutyricum_hallii_Lachnospiraceae_96_8 | 1.5 | 0.023 |  |  |  |  |
| OTU_470_Anaerocolumna_chitinilytica_Lachnospiraceae_94_8 | -3.7 | 0.019 |  |  |  |  |
| OTU_574_Butyricicoccus_pullicaecorum_Oscillospiraceae_96_3 | 9.1 | 0.003 |  |  | 9.3 | 0.003 |
| OTU_624_Blautia_glucerasea_Lachnospiraceae_96 | 4.3 | 0.002 |  |  |  |  |
| OTU_8_Blautia_glucerasea_Lachnospiraceae_99_8 | -2.5 | 0.003 |  |  |  |  |
| OTU_247_Petroclostridium_xylanilyticum_Oscillospiraceae_87_3 |  |  | 4.6 | 0.035 |  |  |
| OTU_43_Clostridium_fessum_Clostridiaceae_98_3 |  |  | -1.4 | 0.037 |  |  |
| OTU_628_Pseudoflavonifractor_capillosus_Ocillospiraceae_96_8 |  |  | -2.7 | 0.004 |  |  |
| OTU_18_Turicibacter_bilis_Turicibacteraceae_99_8 |  |  |  |  | 3.7 | 0.033 |
| OTU_196_Guopingia_tenuis_Christensenellaceae_86_9 |  |  |  |  | 4.9 | 0.011 |
| OTU_548_Mediterraneibacter_faecis_Lachnospiraceae_95_8 |  |  |  |  | -2.8 | 0.002 |
| OTU_55_Aristaeella_lactis_Aristaeellaceae_93_1 |  |  |  |  | 9.8 | 0.000 |
| OTU_622_Vescimonas_fastidiosa_Oscillospiraceae_92_3 |  |  |  |  | 2.2 | 0.034 |
| **2^nd^ sampling day 44** | | | | | | |
| OTU_122_Clostridium_saudiense_Clostridiaceae_100 | 4.3 | 0.020 |  |  | 5.2 | 0.004 |
| OTU_154_Anaeroplasma_varium_Anaeroplasmataceae_90_3 | -3.8 | 0.001 |  |  |  |  |
| OTU_21_Bacillus_salis_Bacillaceae_93_5 | 2.5 | 0.013 |  |  |  |  |
| OTU_238_Aristaeella_hokkaidonensis_Aristaeellaceae_92_3 | 1.5 | 0.032 |  |  |  |  |
| OTU_247_Petroclostridium_xylanilyticum_Oscillospiraceae_87_3 | -2.1 | 0.050 |  |  |  |  |
| OTU_273_Anaerosinus_glycerini_Sporomusaceae_89_2 | 22.8 | 2E-24 | 21.6 | 5E-22 | 23.1 | 0.000 |
| OTU_281_Mammaliicoccus_lentus_Staphylococcaceae_100 | 2.7 | 0.013 |  |  |  |  |
| OTU_290_Ethanoligenens_harbinense_Oscillospiraceae_91_9 | -2.3 | 0.050 |  |  |  |  |
| OTU_302_Brachybacterium_atlanticum_Dermabacteraceae_100 | 2.7 | 0.050 |  |  |  |  |
| OTU_326_Corynebacterium_stationis_Corynebacteriaceae_98_8 | 2.2 | 0.050 |  |  |  |  |
| OTU_358_Clostridium_leptum_Clostridiaceae_92_6 | 3.7 | 0.021 |  |  |  |  |
| OTU_402_Jeotgalicoccus_aerolatus_Staphylococcaceae_100 | 3.0 | 0.013 |  |  |  |  |
| OTU_502_Brevibacterium_senegalense_Brevibacteriaceae_100 | 4.0 | 0.029 |  |  |  |  |
| OTU_509_Guopingia_tenuis_Christensenellaceae_85_9 | -1.0 | 0.050 |  |  |  |  |
| OTU_56_Flintibacter_hominis_Gammaproteobacteria_incertae_sedis_91_6 | 2.5 | 0.050 | 2.6 | 0.036 |  |  |
| OTU_580_Fumia_xinanensis_Oscillospiraceae_92_5 | 3.5 | 0.021 |  |  |  |  |
| OTU_62_Staphylococcus_schweitzeri_Staphylococcaceae_100 | 2.6 | 0.050 |  |  |  |  |
| OTU_622_Vescimonas_fastidiosa_Oscillospiraceae_92_3 | 1.6 | 0.050 |  |  |  |  |
| OTU_103_Butyricicoccus_porcorum_Oscillospiraceae_98_5 |  |  | -10.8 | 0.036 |  |  |
| OTU_129_Clostridium_polysaccharolyticum_Clostridiaceae_94_5 |  |  | 1.2 | 0.029 |  |  |
| OTU_143_Vallitalea_okinawensis_Vallitaleaceae_86_8 |  |  | 2.2 | 0.030 |  |  |
| OTU_161_Aristaeella_hokkaidonensis_Aristaeellaceae_93_1 |  |  | -1.1 | 0.025 |  |  |
| OTU_177_Pseudoflavonifractor_capillosus_Ocillospiraceae_97_3 |  |  | -3.9 | 0.008 |  |  |
| OTU_488_Blautia_argi_Lachnospiraceae_97_5 |  |  | -2.3 | 0.008 |  |  |
| OTU_544_Mediterraneibacter_hominis_Lachnospiraceae_96_8 |  |  | 1.6 | 0.029 |  |  |
| OTU_76_Gemmiger_gallinarum_Oscillospiraceae_98 |  |  | 2.3 | 0.029 |  |  |
| OTU_77_Copranaerobaculum_intestinale_Erysipelotrichaceae_92_7 |  |  | -1.6 | 0.029 |  |  |


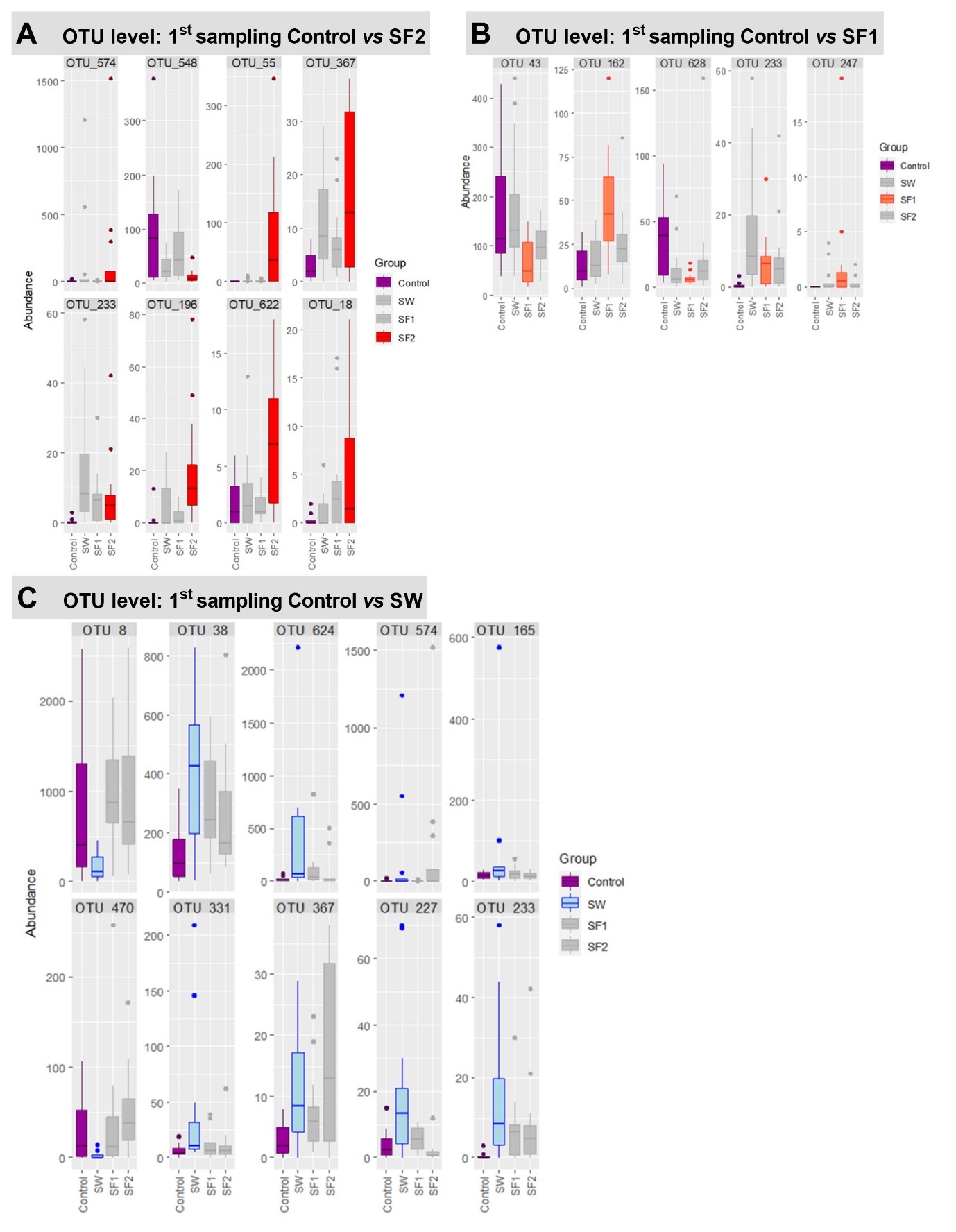


Figure S7. Abundance of cecum microbiota members at OTU level with statistically significant differences in abundance in treatment groups **(A)** SF2 (spores in feed at concentration 10^9^ spores/kg of feed), **(B)** SF1 (spores in feed at concentration of 10^6^ spores/kg of feed) and **(C)** SW (*B. subtilis* PS-216 spores drinking water at the concentration of 10^9^ spores/L) compared to the untreated control, at first sampling (23 days).


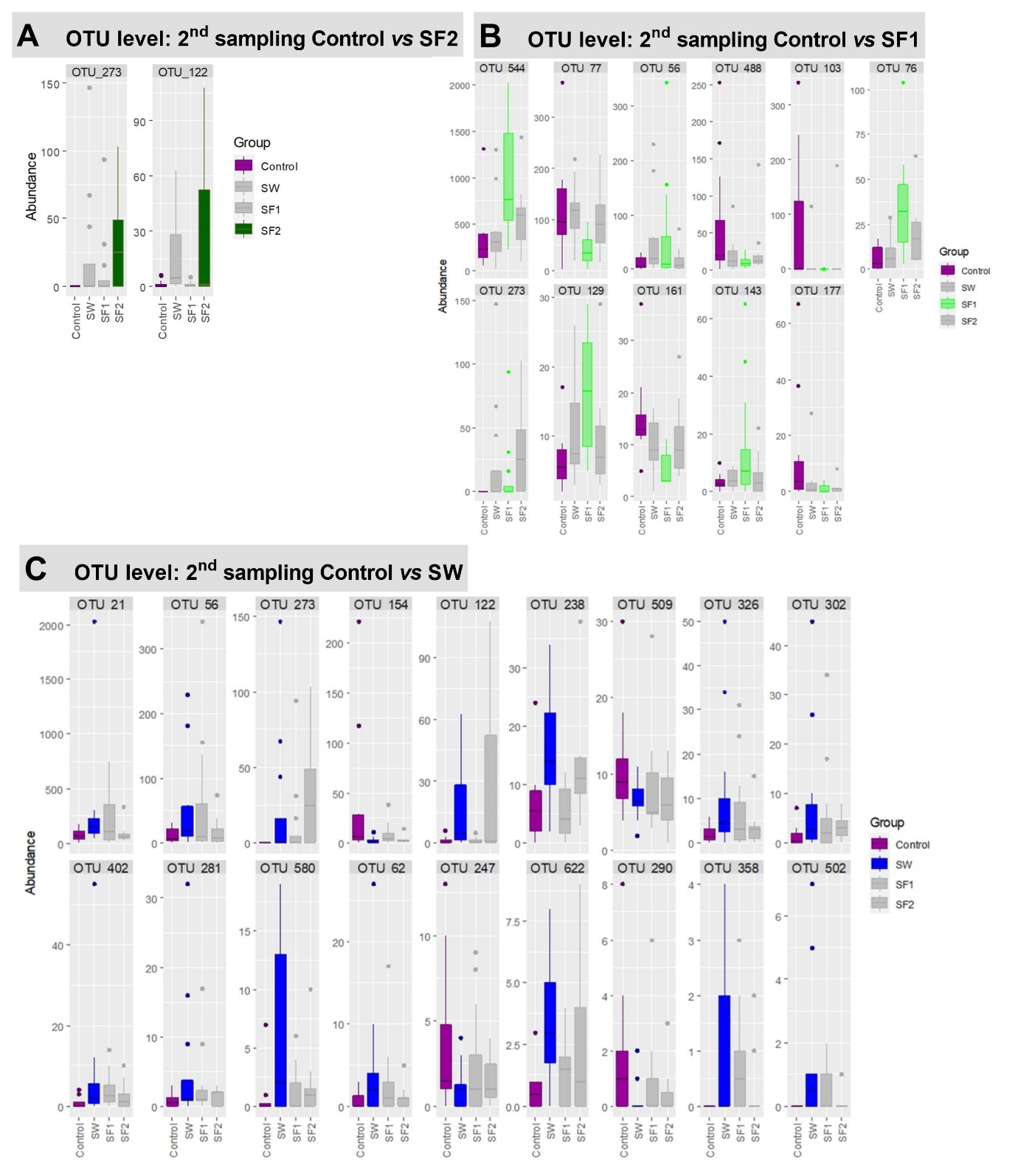


Figure S8. Abundance of cecum microbiota members at OUT level with statistically significant differences in abundance in treatment groups **(A)** SF2 (spores in feed at concentration 10^9^ spores/kg of feed), **(B)** SF1 (spores in feed at concentration of 10^6^ spores/kg of feed) and **(C)** SW (*B. subtilis* PS-216 spores drinking water at the concentration of 10^9^ spores/L) compared to the untreated control, at second sampling (44 days).


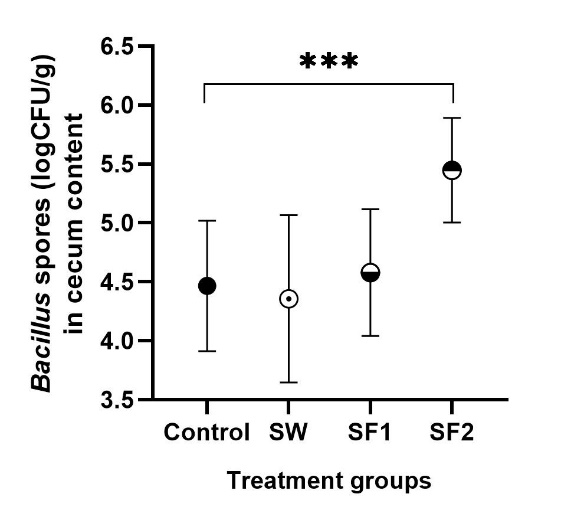


Figure S9. Spore concentration (mean ± standard deviation logCFU/g) in cecum content of broiler chickens untreated control and treated with *B. subtilis* PS-216 spores in drinking water at the concentration of 10^9^ spores/L (SW), in feed at concentration of 10^6^ spores/kg of feed (SF1), and 10^9^ spores/kg of feed (SF2) at second sampling at 44 days of age. Statistical significance is determined using ANOVA with Dunnett’s multiple comparisons test and presented with ***p<0.001.
